# Review of Anodic Tantalum Oxide Nanostructures: From Morphological Design to Emerging Applications

**DOI:** 10.1021/acsanm.4c02000

**Published:** 2024-06-18

**Authors:** Biswaranjan D. Mohapatra, Grzegorz Dariusz Sulka

**Affiliations:** Department of Physical Chemistry & Electrochemistry, Faculty of Chemistry, Jagiellonian University, Gronostajowa 2, 30-387 Krakow, Poland

**Keywords:** Anodization, Metal oxide nanostructures, Anodic Ta_2_O_5_ nanostructures, Surface modification, Growth mechanism, PEC water splitting, Supercapacitors, Corrosion protection, Biomaterials

## Abstract

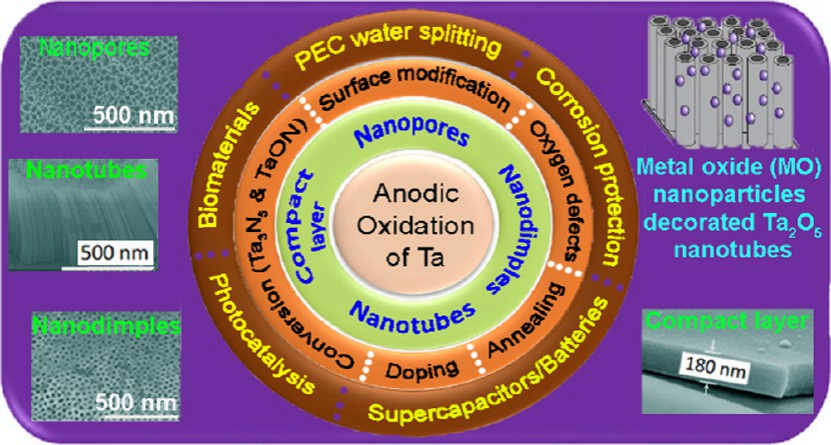

Anodization of transition metals, particularly the valve metals (V, W, Ti, Ta, Hf, Nb, and Zr) and their alloys, has emerged as a powerful tool for controlling the morphology, purity, and thickness of oxide nanostructures. The present review is focused on the advances in the synthesis of micro/nanostructures of anodic tantalum oxides (ATO) in inorganic, organic, and mixed inorganic–organic type electrolytes with critically highlighting anodization parameters, such as applied voltage, current, time, and electrolyte temperature. Particularly, the growth of ATO nanostructures in fluoride containing electrolytes and their applications are briefly covered. The details of the current– or voltage–time transient and its relation to the growth of the anodic oxide films are presented systematically. The main discussion revolves around the incorporation of various electrolyte species into the surface of ATO structures and its effects on their physicochemical properties. The latest progress in understanding the growth mechanism of nanoporous/nanotubular ATO structures is outlined. Additionally, the impact of annealing temperature (ranging from 400–1000 °C) and atmosphere on the crystalline structure, morphology, impurity content, and physical properties of the ATOs is briefly described. The common modification methods, such as decorating with other transition metal/metal oxide, heteroatom doping, or generating defects in the ATO structures, are discussed. Besides, the review also covers the most promising applications of these materials in the fields of capacitors, supercapacitors, memristive devices, corrosion protection, photocatalysis, photoelectrochemical (PEC) water splitting, and biomaterials. Finally, future research directions for designing ATO-based nanomaterials and their utilities are indicated.

## 1 Introduction

The development of innovative synthetic strategies to control the shape and size of nanostructured transition metal oxides (TMOs) has always been considered an important area of research.^1−5^ Nanostructured TMOs hold significant importance due to their numerous potential applications, ranging from energy harvesting technologies to biomaterials.^1−5^ Various synthetic procedures, such as hydrothermal, solvothermal, sol–gel, vapor phase deposition, chemical vapor deposition (CVD), electrodeposition, and electrochemical anodization, can be employed to fabricate nanostructures of TMO.^1,2^ However, among all of these synthetic strategies, electrochemical anodization is considered a simple, cost-effective, and scalable method for producing TMO nanostructures with the potential to control their morphology and purity.^2,6^ In particular, the fabrication of oxide nanostructures of various valve metals, e.g., Ti, Nb, Ta, Hf, and W by anodization has been widely investigated in the last two decades.^7−20^ This method has yielded promising results in various technologically important fields, including hydrogen production, electrochromic devices, corrosion-resistant coatings, solar cells, batteries, sensors, capacitors, catalysts, and biomedical devices.^6−8^ Tantalum oxide (Ta_2_O_5_) characterized by unique properties like high melting point, chemical inertness, and extraordinary refractive index holds significance as a crucial material.^8−14,18^ Tantalum can exist in either the +5 or +4 oxidation state, depending on the oxide phase, i.e., Ta_2_O_5_ and TaO_2_, respectively.^12−14^ However, Ta_2_O_5_ is considered thermodynamically the most stable state. The fabrication of Ta_2_O_5_, particularly by the anodization method, has been intensively investigated in different inorganic (H_2_SO_4_, H_3_PO_4_, NH_4_F, Na_2_SO_4_ solutions), organic (oxalic acid, glycerol, ethylene glycol (EG)), and mixed inorganic–organic electrolytes (H_2_SO_4_ + EG + NH_4_F) at various operating voltages (typically ranging from 10 to 200 V).^13,14,18^ The structure of anodic tantalum oxide (ATO) layers can vary from 0D to 3D, depending on the applied anodizing conditions, such as anodization voltage/current, anodization time, temperature, and pH of the electrolyte. Diverse morphologies, including compact layers, ordered porous structures, nonordered porous structures, nanotubes, and coral-like structures, have been observed in ATOs.^18^

Over the past two decades, continuous research efforts have sought to understand the anodic growth mechanism of nanoporous/tubular metal oxide structures.^15−20^ Typically, electrolytes containing fluoride ions with a suitable anodizing potential range are considered to promote the growth of nanoporous/tube structures, as observed for many valve metals, including Ti, Ta, and Zr.^13−20^ It is noteworthy that the formation mechanisms associated with nanoporous/tube structures of anodic titania (TiO_2_) and Ta_2_O_5_ have been intensively investigated in the previous decade. Various sophisticated analytical techniques, such as X-ray photoelectron spectroscopy (XPS), energy dispersive X-ray (EDX) mapping, and radioactive labeling methods, have been applied to understand the growth mechanisms.^15−20^ The process of nanopores or nanotubes formation involves a competition between the rate of formation of the oxide layer at the metal/oxide interface and the dissolution of the oxide layer at the oxide/electrolyte interface, which is well documented and can be found in reviews by G. D. Sulka, N. L. S. Ngadiman et al., D. Regonini et al., and X. Zhou et al.^15,17−20^

Ta-based materials have been extensively explored not only in the field of energy conversion and storage but also in diverse sectors, including corrosion resistant coatings and osseointegration applications.^21−33^ Particularly, Ta_2_O_5_ nanostructures prepared by anodization can serve as a starting material to produce TaON, TaS_2_, Ta_3_N_5_ nanostructures with promising activity toward photoelectrochemical (PEC) water splitting.^21−25^ Numerous studies have investigated surface doping or functionalization with various metals or nonmetals (e.g., B, Ti, Ni, and Co) and surface decoration with plasmonic materials like gold particles. These endeavors aim to tune the band gap energy for effective photo or photoelectrochemical catalysis.^24,25,34,35^ Additionally, many studies reported the incorporation of various electrolyte species (based on e.g., F, P, and S elements) into the anodic layers of Ta_2_O_5_, introducing new physicochemical properties that may broaden their range of applications.^36−39^ Efforts have also been directed toward creating defects in anodic Ta_2_O_5_ films to modify their physical properties.^40^ For example, S. Xia et al.^40^ demonstrated a lithium capacity of ∼480 mAh g^–1^ with cycling stability over 8000 cycles by inducing oxygen deficiency in the Ta_2_O_5_ matrix. Therefore, different modification methods hold the potential to enhance the properties of anodic tantalum oxide, offering diverse avenues to study these effects.

In this paper, we systematically review the progress in the synthesis of doped and undoped structures of anodic tantalum oxides, post modification methods, and their characterization techniques. We explain how various types of oxide morphologies are generated under the influence of an electric field, consolidating dispersed information in one place and indicating possible future research directions relevant to anodic tantalum oxide structures.

## 2 Tantalum Substrates for Anodization

The methods of preparation of Ta substrates, along with their dimensions and purity, may vary depending on the research objectives and applications. In general, high purity of tantalum (∼99.9%) in the form of foil/sheet/disc/rod is an excellent choice for anodization experiments.^21,22,32,41^ Apart from that, Ta sputter-deposited or coated on glass/Si-wafer/metal substrates is commonly used and documented in various publications.^31,35^ A detailed list of the Ta substrates, including their dimensions and purity, can be found in Table 1.

**Table 1:** Characteristic of Ta Substrates Used for Anodization

Ta substrates	Purity of Ta	Thickness	Ref
Ta disc	99.99%	NA	(28, 41)
Ta disc	99.99%	0.25 mm	(27)
Ta film	99.9%	300 μm	(22, 42)
Ta foil	99.5%	0.1 mm	(43)
Ta foil	99.5%	0.2 mm	(44−46)
Ta foil	99.9%	0.1 and 2 mm	(47)
Ta foil	99.9%	0.1 mm	(24, 25, 40, 48)
Ta foil	99.9%	0.5 mm	(26, 33, 38, 49−51)
Ta foil	99.95%	0.025 mm	(52)
Ta foil	99.95%	0.127 mm	(53−56)
Ta foil	99.95%	0.25 mm	(57−60)
Ta foil	99.96%	NA	(21, 61−64)
Ta foil	99.99%	0.15 mm	(65)
Ta foil	NA	0.127 mm	(66)
Ta, Nb and Ti foil	NA	0.5 mm	(67)
Ti, Zr, Nb, Ta foil	99.95% (Ta, Nb, Ti), 99.60% (Zr)	0.05 mm (Ti, Zr, Nb), 0.1 mm (Ta)	(29)
Ta rod	97%	NA	(68)
Ta rod	99.9%	NA	(32)
Ta sheet	NA	NA	(69)
Ta sheet	99.9%	NA	(70−73)
Ta sheet	99.9%	1 mm	(30, 36, 74)
Ta sheet	99.99%	1.5 mm	(75)
Ta wire	NA	NA	(76)
Ta deposited thin films on Ta	99.95%	200 nm	(35)
Ta particles (size of 0.3 μm) on Ta foil (50 μm thickness)	NA	25 μm	(77)
Ta sputter-deposited on glass	NA	∼280 nm	(78)
Ta sputter-deposited on glass and Al sheet	NA	NA	(79)
Ta on Si wafer	NA	300 nm	(80)
Ta sputter-deposited on n-type Si wafer	96–97%	0.1 μm	(81)
Ta on (100) n-type Si wafer	NA	2–20 nm	(82)
Ta on Mo/Ta plate	NA	∼2 mm	(83)
Ta coated Ti alloy (Ti64)	NA	NA	(31)
Ta–Ti deposited on Si-wafer	99.99%	NA	(84)
Ta–Ti–Pt-TaN film on p-type Si or B-doped Si wafer	NA	220 nm	(85)
Ta foil covered with ∼2 μm thick Al	99.9%	0.1 mm	(23)
Ta foil coated with 2 μm thick Al	NA	0.1 mm	(86)
TaN_*x*_ and Ta_2_Si films sputter-deposited on Si wafers	NA	10–450 nm	(87, 88)
TaN_*x*_	NA	NA	(89)

## 3 Pretreatment of Tantalum Substrates before Anodization

The pretreatment of tantalum substrates before anodization is an essential step to create a uniform, smother, and contaminant-free surface.^32,33,38^ In general, commercially available Ta substrates (e.g., foil/sheet/rod/disc) are coated with grease or oil to protect them from corrosion or aerial oxidation.^43,47,69^ The process of degreasing the metal surface involves ultrasonication of the substrate in a mixture of acetone and ethanol for few minutes, followed by washing with distilled water and drying in a vacuum or N_2_ flow.^43,47^ Subsequently, various polishing methods (mechanical, chemical, and electrochemical) may be employed to remove the surface contaminants and oxides formed by areal oxidation. Mechanical polishing techniques may include rubbing the metal surface with various abrasive papers, such as emery papers (grade 2400) or SiC sandpaper of various grades, such as P500, P600, P800, P1200, and P2500 etc.,^30,33,25^ or polishing with a cloth coated with a diamond paste (1, 0.5, 0.25, and 0.125 mm) or alumina suspension slurries (0.1–0.05 μm particle size).^26,43,47,69^ These steps are crucial for minimizing the roughness and achieving a mirror finish to the metal surface. The details of chemical and electrochemical polishing of Ta substrates are collected in Table 2 and 3, respectively.

**Table 2:** Conditions for the Chemical Polishing of Ta Substrates

Composition of solution	Temp.	Time	Observation/remarks	Ref
Boiling H_2_O	100 °C	1 min	NA	(72)
Dilute NaOH	NA	1 h	NA	(72)
Molten NaOH	NA	Few seconds	NA	(65)
1 M H_2_SO_4_	NA	5 min	To dissolve the air-formed oxide film on the surface	(90)
4 M H_2_SO_4_	NA	2 min	For chemical etching of the surface	(44)
Concentrated HF and 400 g dm^–3^ NH_4_F	NA	2 min	Followed by rinsing in boiling double-distilled water for 5 min to minimize any fluoride contamination	(75)
4 M H_2_SO_4_ and 1 M HF	NA	120 s	For surface chemical etching	(32)
H_2_SO_4_ (95%):HNO_3_ (70%):HF (48%) (5:2:1.5 vol %)	NA	Few seconds	NA	(69)
H_2_SO_4_ (95%):HNO_3_ (70%):HF (48%) (5:2:2 vol %)	NA	Few seconds	To remove all scratches and any grease or other foreign materials	(70)
H_2_SO_4_ (96%):HNO_3_ (70%):HF (48%) (5:2:2 vol %)	25 °C	NA	NA	(79)
H_2_SO_4_ (98%):HNO_3_ (70%):HF (40%) (5:2:1.5 vol %)	NA	NA	NA	(72)
H_2_SO_4_ (98%):HNO_3_ (70%):HF (48%) (5:2:2 vol %)	NA	10 s	NA	(73)
H_2_SO_4_:HNO_3_:HF (5:2:2 vol %)		15 s	Followed by chemical polishing in NH_4_F (300 g dm^–3^) in HF for 5 min	(61)
HF:HNO_3_:H_2_O (1:2:7 vol %)	NA	30 min	NA	(40)
H_2_SO_4_ (95–98%):HNO_3_ (65–68%):HF (48%):H_2_O (17:7:7 69 vol %)	NA	NA	The polishing was conducted in the ultrasonic bath	(46)

**Table 3:** Conditions for Electrochemical Polishing of Ta Substrates

Composition of solution	Temp.	Applied current density/potential	Time	Observation/remarks	Ref
Concentrated H_2_SO_4_:HF (90:10 vol %)	NA	100 mA cm^–2^	10 min	NA	(75)
H_2_SO_4_ (95–98%):HF (48%) (9:1 vol %)	NA	15 V	5 min	The solution was stirred during polishing	(91)
1 M H_2_SO_4_	RT	25 V	10 min	The solution was stirred during polishing	(33, 38)

## 4 Anodization of Tantalum

**Figure 1:**
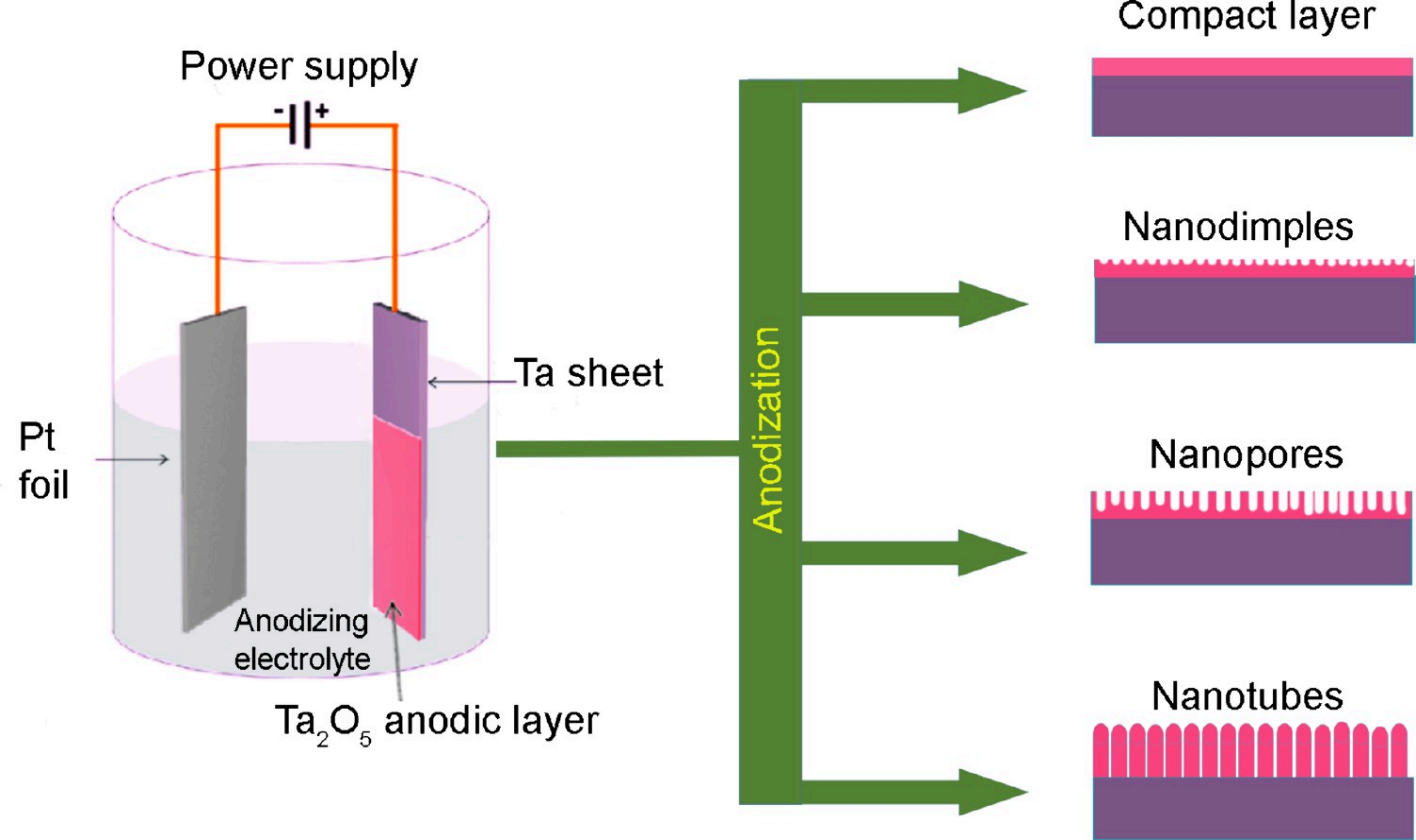
Pictorial representation showing the fabrication of ATOs with various morphologies.

The formation of protective or decorative layers of anodic metal oxides through anodization has been conducted for many decades.^15,19,20^ Anodization is generally carried out in an electrochemical cell with a two-electrode configuration, wherein the metal foil undergoing the anodic process is connected to the positive output, and a platinum/conductive carbon plate is connected to the negative output of a power supply.^15−20^ The electrochemical processes leading to the formation of anodic metal oxide structures are initiated by applying a suitable voltage between the two electrodes. Various anodization parameters, like composition of the electrolyte, anodizing voltage/current, time, temperature, and pH of the electrolyte, may determine the final morphology of the anodic oxides.^15−20^ A generalized pictorial representation showing the anodization configurations and the formation of ATO structures is presented in Figure 1.

### 4.1 Different Types of Morphologies of Anodic Ta_2_O_5_

**Figure 2:**
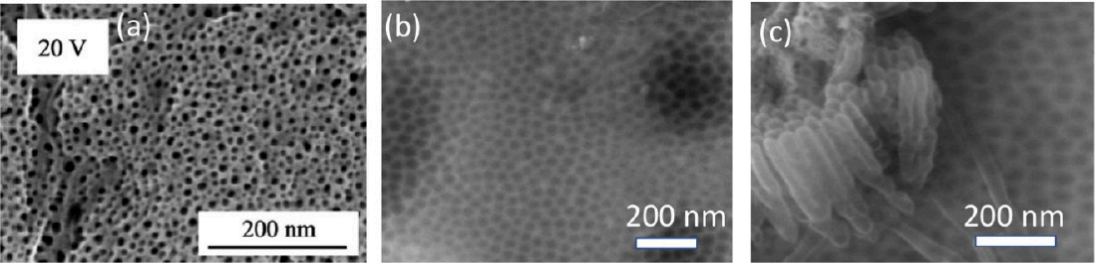
(a) SEM image of Ta anodized by sweeping the potential from *E*_OCP_ to 20 V in 1 M H_2_SO_4_ + 2 wt % HF (scan rate of 10 mV s^–1^). Reproduced with permission from ref (43). Copyright 2006 Springer. (b) SEM images after anodization of polycrystalline Ta surface for 5 s and (c) 10 s in 16.4 M H_2_SO_4_ + 2.9 M HF at 15 V. Reproduced with permission from ref (53). Copyright 2009 American Chemical Society.

The 20th century research on ATO structures was primarily dedicated to the understanding optical interference effects (coloring effect) and capacitance–voltage relations with respect to the formed oxide thickness.^87,92−94^ In most cases, a two-component anodizing solution, comprising various concentrations of H_2_SO_4_, Na_2_SO_4_, H_3_PO_4_, HCl, NaBF_4_, citric acid, etc., in water was utilized for the formation of ATO structures.^61,66,70−73,75,76,87,88,93−95^ The anodizing potential was maintained below the dielectric breakdown potential, typically up to 300 V, resulting in the formation of a uniform, compact, and amorphous ATO layer. In 2005, I. Sieber et al. reported, for the first time, the formation of porous ATO layer by anodizing Ta foil in a 1 M H_2_SO_4_ electrolyte containing a small amount of HF (0.1–3 wt %).^43,47^ The anodizing potential was ramped from *E*_ocp_ to 20 V at 10 mV s^–1^, resulting in an observed ATO layer thickness of about 130 nm (Figure 2a).^43^ The presence of fluoride ions in the electrolyte was found to promote the formation of porous ATO structures. They predicted that during anodization, fluoride ions move inward into the ATO structure about 1.85 times faster than oxygen ions, and the mobility of fluoride ions is independent of the film thickness. The same group observed also a thickening (32 to 130 nm) of porous ATO layer in F^–^ ion-containing electrolytes compared to F^–^ ion-free electrolytes.^43^ It was indicated that the morphology and thickness of the oxide layer depend strongly on the applied potential, scan rate, and HF concentration.

Similarly, ATO layers with nanotube morphology can be fabricated using F^–^ ion-containing electrolytes.^53,96^ H. A. El-Sayed et al.^53^ demonstrated the formation of nanodimple and nanotube morphology of ATOs in a stirred mixture of concentrated HF and H_2_SO_4_ (Figure 2b,c). The stirring of electrolytes during anodization is considered an important condition to obtain a well-patterned surface structure; otherwise, a smooth surface may be obtained. Subsequent studies indicated the dependence of the H_2_SO_4_ to HF ratio to achieve ordered nanoporous/nanotube structures of ATO.^91,97,98^ Details of the anodizing conditions, including the composition of the electrolyte, potential/current, time, and other factors influencing the formation of compact or nanoporous or nanotubular structures, can be found in Table 4.

**Table 4:** Selected Procedures of Ta Anodization along with the Observed Morphology of the Anodic Film, Post-treatment Conditions, and Applications^a^

Electrolyte	Voltage/current density	Anodizing time (min)	AT (°C)	ATO morphology	Anodic layer thickness (μm)	Post-treatment	Applications	Ref
0.1 wt % Na_2_SO_4_	2 and 8 mA cm^–2^, and 100 V	Up to 166.66	0–88	Compact layer	0.02–0.5	Annealing in vacuum at 500–800 °C	NA	(70)
0.1 wt % Na_2_SO_4_	50–460 V	Up to 16.66	NA	Compact layer	NA	NA	NA	(71)
0.1 M Na_2_SO_4_/0.1 M H_3_PO_4_/	250 mV s^–1^ until ≤13 V	60	RT	Compact layer	∼0.006–0.015		NA	(85)
0.1 M Na_2_SO_4_/1 M KCl/0.1 M H_3_PO_4_	70 V		NA	NA	NA	NA	NA	(39)
0.1 M solution of (NH_4_)B_5_O_8_/ Na_2_WO_4_/Na_4_SiO_4_/Na_2_MoO_4_/Na_2_CrO_4_/ Na_3_VO_4_/H_2_SO_4_/H_3_PO_4_ 1 M solution of NH_4_F/KCl/KBr/KI	1 mA cm^–2^ to 100 V	NA	25	Compact layer	NA	NA	NA	(75)
1.15 M Mg(CH_3_COO)_2_/0.5 M Ca(H_2_PO_2_)_2_ + 1.15 M Ca(HCOO)_2_	galvanostatic (150 mA cm^–2^) up to 200, 300, 400, and 500 V	5 and 10	NA	Micro and nanoporous layer	NA	NA	Biomaterial/bone implantation	(32)
0.1 M sodium citrate/0.1 M potassium sodium tartrate/0.1 M sodium adipate/0.1 M NaOH	50 V	166.66	NA	Compact layer	NA	NA	Tantalum-based capacitors	(68)
0.1 wt % (0.01 M) H_2_SO_4_	75 V, up to 1 mA cm^–2^	60	34	Compact layer	NA	NA	NA	(73)
0.05 M H_2_SO_4_	1 mA cm^–2^	NA	NA	Compact layer	NA	Annealing in air at 300–400 °C	Capacitors	(95)
0.5 M H_2_SO_4_	0.7 mA cm^–2^	1.66–10	NA	Compact layer	NA	Annealing in air at 300–800 °C	NA	(41)
0.5 M H_2_SO_4_	20 ± 0.1 A cm^–2^	0.05–10	25 ± 0.5	Compact layer of Ta_2_O_5_ on Ta/Mo plate	∼0.15	NA	Corrosion resistance behavior	(83)
0.05–0.1 M H_2_SO_4_	Up to 100 V at about 10 mA cm^–2^	NA	NA	Compact layer	NA	NA	NA	(72)
0.05 M H_2_SO_4_/40% H_2_SO_4_/100% H_2_SO_4_	1.0 mA cm^–2^ up to 15 V	0.33 and 0.66	2–50	Compact layer	NA	NA	NA	(65)
0.5 M H_2_SO_4_/0.5 M H_3_PO_4_	10–100 V	NA	NA	Compact layer	0.03–0.22	NA	NA	(99)
0.05–9 M H_2_SO_4_/1.3–11.7 M HCl/4 M NaBF_4_,	Up to 40 V, 0.2 mA cm^–2^	Up to 11	NA	Compact layer	NA	NA	NA	(69)
1 M H_2_SO_4_/1 M H_3_PO_4_	5 and 20 V, and −1.0 to 5.0 V vs SCE (50 mV s^–1^)	60	NA	Compact and porous oxide layers	∼0.07	NA	Biomaterial/biocompatibility	(30)
2 M H_2_SO_4_	105–200 V	1	NA	Island-like microstructure	NA	Soaked in simulated body fluid	Bioactivity	(90)
5 M H_2_SO_4_/ 0.002–5 M H_3_PO_4_/0.05–2 M H_2_CrO_4_/0.2 M citric acid	Up to 50 V	NA	NA	Compact layer	NA	Annealing in vacuum at 450 °C	NA	(95)
H_3_PO_4_	5–300 A m^–2^	NA	NA	Compact layer	NA	NA	NA	(61, 63)
0.01 wt % H_3_PO_4_	Up to 300 V or scintillation voltage	NA	20 ± 0.2		NA	NA	NA	(61)
0.0001 M H_3_PO_4_	1, 3, and 8 V (5 mV s^–1^)	NA	NA	Nanoparticles 25–23 nm	NA	NA	Capacitors	(77)
0.0008–0.8 M H_3_PO_4_	0–350 V	10	NA		NA	NA	NA	(100)
0.01–10% H_3_PO_4_/0.01% H_3_PO_4_ + 0.1–0.15% citric acid	0.04 Ag^1–^, 100–300 V	10	85	Compact layer	NA	NA	Capacitors	(93)
0.1 M H_3_PO_4_/0.1 M oxalic acid	30–70 mA cm^–2^	0.16–10	10–32	Compact layer	0.3–0.8	NA	Galvanoluminescence materials	(56)
0.5 M H_3_PO_4_	5–20 mA/cm^2^ 10–50 s	0.166–0.83	NA	NA	NA	Annealing in air at 500–700 °C	NA	(81)
0.5 M H_3_BO_3_	0–650 V (scan rate 0.1 V s^–1^) and at 650 V	60	RT	Nanorods D = 60 ± 10 nm	∼600 nm	Annealing in NH_3_ at 1000 °C	PEC water splitting	(23)
0.5 M H_3_BO_3_	0–650 V (0.1 V s^–1^) and kept at 650 V for 1 h	60	RT	Nanorod arrays D = 180 nm	NA	Annealing in NH_3_ at 1000 °C and then modified with cobalt-phosphate	PEC water splitting	(86)
1 M CH_3_COOH + 1 M H_3_PO_4_/1 M CH_3_COOH + 1 vol % HF/1 M CH_3_COOH + 1 M H_3_PO_4_ + 1 vol % HF	30 V	30	30 ± 2	Compact layer	NA	NA	NA	(64)
0.01 wt % citric acid	5–230 V	60	NA	NA	0.01 to 0.45	NA	Capacitors	(87, 88)
0.5 wt % citric acid	1.0 mA cm^–2^	NA	25	Compact layer	NA	NA	NA	(80)
5 wt % citric acid	10, 20, 40, and 80 mA cm^–2^	5	30	Compact layer	NA	NA	NA	(101)
0.01, 0.05, 0.1, 0.2 M citric acid	Up to 2 V (vs Ag/AgCl) and 0.1 mA/cm^2^	30	NA	Compact layer	0.004	NA	NA	(102)
0.1 M citrate buffer	Up to 7 V (scan rate = 100 mV s^–1^)	NA	RT	Compact layer	NA	NA	Study of memristive effects	(80)
1 M H_2_SO_4_ + 0.1–3 wt % HF	*E*_OCP_ to 20 V (scan rate: 10 mV s^–1^)	NA	NA	Nanoporous layer D = 5–35 nm	0.16 to 0.8	NA	NA	(47)
1 M H_2_SO_4_ + 2 wt % HF	*E*_OCP_ to 10–20 V (scan rate: 10–100 mV s^–1^)	NA	NA	Nanoporous layer D = 2–10 nm	0.032–0.13	NA	NA	(43)
NH_4_F/HF + H_2_SO_4_	20 to 100 V	5	NA	Nanodimples and nanopores D = 6–27 nm	NA	NA	NA	(97)
H_2_SO_4_:HF (24:1 vol %)	10–200 V	60	NA	Nanodimples D = 38–190 nm	NA		Bioactivity (cell proliferation and osteogenic differentiation)	(46)
H_2_SO_4_ (95–98%):HF (48) (9:1 vol %)	10–20 V	10	NA	Nanodimples D = 30–50 nm	NA	NA	NA	(96)
H_2_SO_4_ (95–97%):HF (38–40%) (9:1 vol %)	30 V	5–20	NA	Nanopores D = 25–65 nm	NA	NA	Biomaterial applications/interaction with cells	(55)
H_2_SO_4_ (95–98%):HF (48%) (9:1 vol %)	15 V	10	RT	Nanodimples D = 55–60 nm	NA	NA	NA	(91)
H_2_SO_4_ (95–98%):HF (48%) (9:1 vol %)	15 V	0.08–2	∼23	Nanodimples Nanotubes D = 20–25 nm	0.25–2.7	NA	NA	(53)
H_2_SO_4_ (85%):HF (48%) (25:1 vol %)	30 V	10	NA	Nanotubes D = 60–70 nm	10–13	Annealing in H_2_S at 625 °C	Superconductivity	(22)
H_2_SO_4_ (95–98%):HF (40%) (90–95:5–10 vol %)	75–175 mA cm^2^	1–4	NA	Nanotubes D = 75–100 nm	3.5–26.5	NA	NA	(74)
H_2_SO_4_ (95–98%):HF (48%) (96:4 vol %)	10–200 V	60	NA	Nanotubes D = 40–270 nm	1–122	NA	NA	(45)
16 M H_2_SO_4_ + (0.2–1.1 M) HF	15 V	0.08–5	NA	Nanotubes	0.14–1	NA	NA	(54)
16 M H_2_SO_4_ + (0.1–1.5 M) HF	15 V	Up to 1.66	NA	Nanoporous/nanotubular oxide layer D = 30–40 nm	∼1.5 μm	NA	N. A	(98)
1 M H_2_SO_4_ + 3.3 wt % NH_4_F	20 V	2	NA	Nanoporous D = 35–65 nm	1.17 ± 0.05	NA	Bioactivity/Ca^2+^ ions absorption	(29)
Borate buffer (0.42 M H_3_BO_3_ + 0.08 M Na_2_B_4_O_7_) (pH = 8.0)	10 and 20 V vs SCE	NA	NA	Compact layer	0.02 and 0.04	NA	Resistive switching memories	(78)
H_2_SO_4_:HF:H_2_O (7–9:0.125–1:0.5–2.5 vol)	15–50 V	0.0833–2	RT	Nanodimples, Nanotubes D = 30–150 nm	0.005–5	NA	NA	(49)
H_2_SO_4_:HF (48%):H_2_O (50:0.1–5:0–15 vol)	10–90 V	20	RT	Nanotubes Inner D = 30–35 nm Outer D = 50–70 nm	1.8–35	NA	NA	(42)
H_2_SO_4_:NH_4_F:H_2_O (85.6:0.8:13.6 wt %)	1.2 A cm^–2^	0.0166–0.0666	NA	Nanotubes D = 100 nm	4.6–10.0	Annealing in NH_3_ at 1000 °C and then modified with cobalt-phosphate	PEC water splitting	(25)
H_2_SO_4_:HF:H_2_O (89.2:2.2:8.6 vol %)	15–250 V	0.333–2	25	Nanotubes D = 50–120 nm	NA	Annealed in O_2_ at 400 °C	Photocatalytic degradation of methylene blue (MB)	(36)
H_2_SO_4_:HF:H_2_O (95:1:4 vol %)	50 V	20	0–50	Nanotubes D = ∼100 nm	3–5	Annealing in air at 550–800 °C	Photocatalytic water splitting	(27)
H_2_SO_4_:HF:H_2_O (95:1:4 vol %)	50 V	20	50	Nanotubes Inner D = ∼50 nm, Outer D = ∼100 nm Nanoparticles D = ∼60 nm	∼5	Annealing in air at 800–1000 °C	Catalytic oxidation of CO	(28)
H_2_SO_4_ (98%):HF (48%):H_2_O (95:1:4 vol %)	50 V	20	50	Nanotubes Inner D = ∼50 nm Outer D = ∼100 nm	∼5	Annealed in air at 450 °C and then decorated with NiO	Photochemical hydrogen production	(34)
H_2_SO_4_ (98%):HF (48%):H_2_O (95:1:4 vol %)	50 V	1–10	RT	Nanotube structure Inner D = ∼80 nm Outer D = ∼170 nm	NA	Annealing in air at 784.85 and 854.85 °C	NA	(50)
H_2_SO_4_ (98%):HF (40%):EG (99:1:5 vol.)	15 V	up to 5	NA	Nanotubes Inner D = 40 nm	1	Annealed in air at 450 °C	Corrosion resistance and bioactivity in simulated body fluids	(31)
45 mL H_2_SO_4_ + 2.5 mL glycerol +2.5 mL H_2_O + 0.5 g NH_4_F	1^st^ step: 60 V, 2^nd^ step: 0–60 V (20 V s^–1^) and 3^rd^ step: 60 V	1^st^ step: 10 3^rd^ step: 1	40, 60, and 80	Nanotubes D = ∼100 nm	4	Annealing in NH_3_ at 1000 °C and then loaded with Co(OH)_*x*_	PEC water splitting	(24)
H_2_SO_4_ (90 vol %) + glycerol (5 vol %) + H_2_O (5 vol %) + 0.27 M NH_4_F	10–20 V	5–10	NA	Nanotubes D = 41 nm	1.68–2.19	Annealed at 500 °C in air/NH_3_/N_2_/H_2_ and then decorated with Bi_2_S_3_	Photocatalytic degradation of toluene	(51)
90 vol % H_2_SO_4_ (95–98%) + 5 vol % diethylene glycol (DEG) + 5 vol % H_2_O + 0.24 M NH_4_F	7–40 V	Up to 3.5	NA	Nanodimples and Nanopores D = 25–60 nm	5 nm	Coated with gold nanoparticles	Photocatalytic conversion of para-aminothiophenol (PATP) to p,p′-dimercaptoazobenzene (DMAB)	(35)
H_2_SO_4_ (98%):HF (48%) (1:9 and 2:8 vol) + 0.05–0.1 M H_3_PO_4_/ EG (5–10 vol %)/ DMSO (5–10 vol %)	10–20 V	10	NA	Nanodimples D = 40–55 nm Nanorods Nanotubes	1.3 10–19	Annealing in air at 300 and 400 °C	NA	(57)
Ethanol (anhydrous, 1% H_2_O)	–0.5 to +4 V vs Ag/AgCl (0.05 V s^–1^)	NA	NA	Nanoparticles 3–5 nm	NA	Decorated with Ni particles	NA	(82)
EG + 0.2 M NH_4_F	15 V	120	50°	Nanoporous layer D = 8–10 nm	NA	annealing at 450 °C in argon atmosphere	Cathode material for microbatteries	(40)
98 vol % EG + 2 vol % H_2_O + 0.3 wt % NH_4_F	20 V	NA	25	Nanopores and nanotubes Outer D = 41.8–62.9 nm Inner D = 30.4 nm	NA	Annealed at 450 °C in Ar	Photocatalytic degradation of MB	(84)
97 vol % EG (99%) + 3 vol % H_2_O + 0.5 wt % NH_4_F	100 V	2	NA	Nanotubes D = 60 ± 5 nm	0.525	Annealing in NH_3_ at 700 °C	PEC water splitting	(21)
97 vol % EG + 3 vol % H_2_O + 0.3–2.4 wt % NH_4_F	40–60 V	20	RT	Compact layer and nanopores D = 20–30 nm	5.3	NA	NA	(38)
97 vol % EG (99.9%) + 3 vol % H_2_O + 2.4 wt % NH_4_F	60 V	20	NA	Nanoporous layer D = 20–40 nm	N. A	NA	Bioactivity/osseointegration implants	(33)
97 vol % EG (99.8%) + 3 vol % H_2_O + 1.2 g L ^–1^ NH_4_F	40 V	20	<40	Compact layer with randomly distributed pores	∼50 nm	Mn_3_O_4_ NP coating	Corrosion resistance behavior	(44)
50 mL EG + 0.5–3 wt % NH_4_F + 0–6 vol % H_2_O+ 0–1 vol % H_2_SO_4_	20 V	60	NA	Compact layer and nanotubes	NA	Annealed at 500 °C in air	NA	(59)
50 mL EG + 1–3 wt % NH_4_F + 2–10 vol % H_2_O + 0–0.5 vol % H_2_SO_4_	20 V	30 and 90	RT	Nanotubes D = 15–20 nm	NA	Annealing at 440 and 550 °C in air	UV sensor	(58)
50 mL EG + 0.675 g NH_4_F + 2 mL of H_2_O + 0.25 mL H_2_SO_4_	20 V	30 and 60	NA	Nanotubes D = 10–50 nm	NA	NA	Humidity sensor	(60)
EG + 3% NH_4_F + 10% H_2_O + 0.25% H_3_PO_4_	20 V	60	NA	Coral-like structures with nanopores D = 30–50 nm	0.15	Annealing in air at 550 °C	Photocatalytic degradation of phenol	(26)
Glycerol (99.8%, anhydrous) + 10 wt % K_2_HPO_4_	2–50 V	up to 26.66	150–200	Nanoporous D = ∼25 nm	1.5–90	NA	NA	(48)
Glycerol +0.1–0.5 M NH_4_F + 0.1 wt % H_2_O	10–50 V	18	NA	Nanopores D = 10–40 nm	Up to 15	NA	NA	(62)
2.0 mL H_2_O (non ^18^O or 10 at. % ^18^O) + 3 wt % ammonium citrate + biological solutions (urine and blood serum and plasma)	10 mA cm^–2^	Up to 10	22 ± 1	NA	NA	NA	NA	(52)

a D – diameter, L – length, AT – anodizing temperature, EG – ethylene glycol, DMSO – dimethyl sulfoxide, PEC – photoelectrochemical, MB – methylene blue.

### 4.2 Incorporation of Species from Anodizing Electrolytes

In this section, we will delve into significant contributions from the 20^th^ century to illuminate the earlier understanding of the growth mechanism of anodic tantalum oxides as well as their morphologies and physical properties. The pioneering work of D. A. Vermilyea in 1953^70^ marks the earliest exploration of anodic oxidation of Ta and growth of tantalum oxide at electrolyte/oxide. In this study, both theoretical and experimental attempts were made to understand the rate of growth of tantalum oxide films under various anodizing conditions. The relations among the rate of oxide growth, electric field, and temperature were established. Notably, a growth rate of 5.24 nm s^–1^ and 0.05 nm s^–1^ was observed at the anodizing current density of 10 mA cm^–2^ and 0.1 mA cm^–2^, respectively, in 0.1 wt % Na_2_SO_4_ at 0 °C. Furthermore, this investigation demonstrated variations in the oxide film thickness as a function of voltage and time at different anodizing currents. The study also successfully correlated the rate of oxide formation with heat generated at the oxide layer through the passage of current. X-ray and electron diffraction studies revealed that the oxide films formed in this study were amorphous Ta_2_O_5_ with the capability of crystallization through annealing at temperatures at 500–800 °C in a vacuum.

In the following study, D. A. Vermilyea studied the growth of ATO films on both rough and polished tantalum surfaces.^71^ The effect of polishing the tantalum sheet on the surface morphology of ATO films was investigated by optical microscopy. In this study, specimens with deep scratches exhibited no evidence of oxide formation for an anodizing current density up to 2 mA cm^–2^. However, at higher anodizing current densities, tantalum oxide was observed to grow on both scratched and polished tantalum substrates. This study, for the first time, demonstrated the migration of metal ions through the oxide layer during its growth. Subsequent efforts were made to understand the rate controlling step and the movement/migration of metal ions during the anodic oxide growth on Ta.^90,103^ For example, B. Verkerk et al.^103^ investigated the mechanism of tantalum oxide growth utilizing radioactive labeling techniques. In 1972, E. Gunzel^95^ examined the anodization of Ta in electrolytes, such as chromic acid, phosphoric acid, sulfuric acid, and citric acid with variations in concentrations. This study suggested, for the first time, the possibility of incorporation of sulfur and phosphorus from electrolytes into the anodic oxide layer. Moreover, it also indicated the incorporation of organic electrolytes during anodization in citric acid and suggested that these foreign species may influence the oxygen distribution in the oxide layer. Similarly, earlier studies on understanding of ATO formation and the incorporation of electrolyte species into the oxide matrix can be traced back to 1978 with the work of F. Arifuku et al. and later in 1996 by K. Shimizu et al.^69,75^ F. Arifuku et al.^69^ examined the anodization of Ta in various electrolytes, such as phosphoric acid, nitric acid, hydrochloric acid, potassium fluoride, sodium chloride, lithium sodium perchlorate, sodium borofluoride, sodium orthosilicate, sodium hydroxide, sodium sulfate, ammonium sulfate, nickel sulfate, and sodium sulfite. Utilizing the secondary ion mass spectrometry (SIMS) technique, they analyzed the incorporation of electrolyte species into the oxide films during anodization. This study revealed no incorporation of Li^+^, Na^+^, K^+^, B, and N in the oxide layer. In addition, the researcher thoroughly investigated the amount of incorporated elements, such as sulfur, chlorine, and fluorine, as well as the depth of incorporation with variations in the concentration of the corresponding anodizing electrolytes. The study further demonstrated that the amount of incorporated sulfur and chlorine along with the relative depth of incorporation increased with an increase in the concentration of H_2_SO_4_ and HCl. On the other hand, V. E. Borisenko et al.^66^ studied the oxidation of tantalum by anodizing in 0.5 M H_3_PO_4_ followed by thermal annealing. Using Rutherford back scattering (RBS) and SEM techniques, they explored the composition, thickness, and morphology of the oxidized tantalum. This study also disclosed the incorporation of phosphorus (1.6 to 1.8 atom %) into the formed ATO films. Further, J. M. Albella et al.^100^ conducted Ta anodization in an H_3_PO_4_ solution and demonstrated that phosphorus species incorporated into the oxide layer are in the form of PO_4_^3–^ anions.

**Figure 3:**
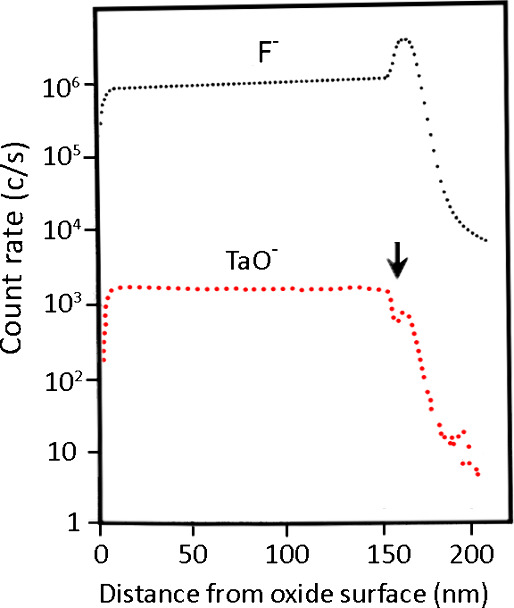
Secondary ion mass spectroscopy (SIMS) depth profile of the film formed on tantalum at 1 mA cm^–1^ (to the voltage of 100 V) in a 1 M ammonium fluoride electrolyte at 25 °C. The decreasing TaO^–^ yield associated with the region of enrichment of fluoride species is indicated by the arrow. Reproduced with permission from ref (75) Copyright 1996 Taylor and Francis.

**Figure 4:**
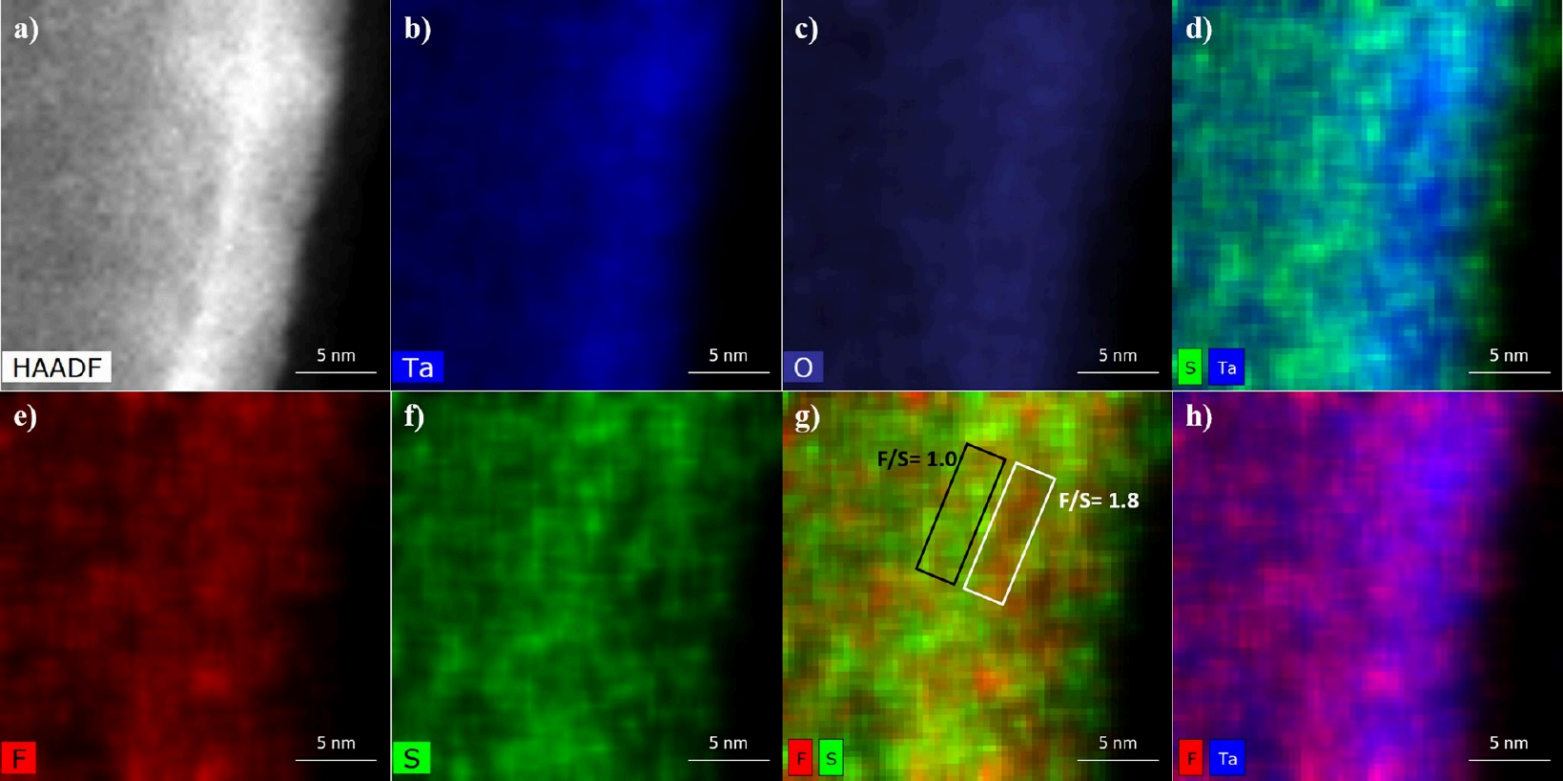
(a) HAADF-STEM and (b–h) EDS spectrum images showing the elemental distribution for the anodic nanotube wall formed in H_2_SO_4_:HF electrolyte (8:0.5 vol %) at room temperature under 15 V for 120 s. The area underlined by the black and white boxes in (g) was used to estimate the F/S ratio using the EDS spectrum of each specific region. Reproduced with permission from ref (49). Copyright 2020 Elsevier.

In a noteworthy investigation, K. Shimizu et al.^75^ explored the incorporation and subsequent mobility and immobility of electrolyte species (cations and anions) into the anodic tantalum oxide layer. Various electrolytes, such as (NH_4_)B_5_O_8_, H_3_PO_4_, H_2_SO_4_, Na_2_WO_4_, Na_4_SiO_4_, Na_2_MoO_4_, Na_2_CrO_4_, Na_3_VO_4_, NH_4_F, KCl, KBr, and KI, at different concentrations, were used as anodizing electrolytes. The SIMS depth profiling technique was employed to examine the electrolyte species incorporated in the tantalum oxide layers. The study demonstrated a relatively uniform distribution of silicon species (Si^–^), PO_4_^3–^ and BO^–^ throughout the outer layer. However, for tungstate, molybdate, chromate, and vanadate electrolytes, the incorporated impurities (W, Mo, Cr, and V) or anion species were suggested to be present only in the outermost layer, with the thickness too small to be determined by SIMS. Utilizing the XPS technique, the authors revealed that the oxy-anions of various metals, e.g., B, W, Mo, V, and Cr, may dissociate to release corresponding cation species (B^3+^, W^6+^, Mo^6+^, V^5+^, and Cr^6+^) which migrate outward at different rates. For the electrolytes containing halide ions, it was observed that Cl^–^, Br^–^, and I^–^ ions may not be incorporated into the oxide structure, whereas F^–^ ions can be incorporated into the growing oxide layer and migrate inward at a faster rate in comparison to that of the O^2–^ ions. As a result, they accumulate at the metal/oxide interface, forming a distinct layer of TaF_5_ approximately 5 nm thick (Figure 3).^75^ The existence of this layer at the metal-oxide interface, reported for the first time, has significant implications, particularly in terms of diminished adhesion between the oxide film and the metal substrate. It was assumed that a higher ionic radius of Cl^–^, Br^–^, and I^–^ ions when compared to oxygen ion is the main reason behind their nonincorporating behavior. Subsequently, advanced analytical techniques, such as high-angle annular dark-field scanning transmission electron microscopy (HAADF-STEM) and EDS mapping, were employed to investigate this oxide doping effect. Figure 4 shows the HAADF-STEM and EDS mapping images of an ATO nanotube wall formed in a mixture H_2_SO_4_:HF (8:0.5 vol %) at 15 V, which clearly indicates a random distribution of F and S in the surface layer of the ATO nanotube.^49^ Recent studies by M. M. Momeni et al.^36,37^ have corroborated the presence of F and S in ATO nanotubes prepared in an H_2_SO_4_ + HF + H_2_O electrolyte. Additionally, similar findings regarding F incorporation were also observed in anodizing electrolytes of an aqueous–organic mixed type. L. Fialho et al.,^38^ through EDX and XPS studies, demonstrated the presence of F^–^ ions in the ATO layer synthesized in the electrolyte based on EG + H_2_O + NH_4_F.

From a practical standpoint, it is anticipated that the incorporation of electrolyte species and/or impurities in the anodic layer can significantly modify its physical properties. For example, the incorporation of phosphate in titanium or tantalum oxide layer may alter the crystallization temperature of the amorphous anodic oxide layer, enhance the breakdown strength of the material, or can reduce the dielectric constant of the material.^81,85,99,104^ J. R. Sloppy et al.^85^ showcased the superior properties of phosphate incorporated ATO layers for use as a dielectric material in electrolytic capacitors. Therefore, the impact of anodizing electrolyte composition on the impurity level in the ATOs and, consequently, their properties is a topic of discussion that warrants further attention.

### 4.3 Effect of Electrolyte Composition

The composition and concentration of anodizing electrolytes significantly affect the morphology of ATO layers.^15−20^ Electrolytes used for anodization of Ta can be broadly classified into three categories: (i) inorganic including, e.g., H_2_SO_4_, Na_2_SO_4_, H_3_PO_4_, HCl, NaBF_4_, H_2_CrO_4_, etc., (ii) organic acids, such as citric acid, oxalic acid, acetic acid, and (iii) mixtures of inorganic and organic electrolytes.^21−105^ Earlier studies on anodization of Ta in two component electrolytes, e.g., H_2_SO_4_, Na_2_SO_4_, H_3_PO_4_, HCl, NaBF_4_, chromic acid, citric acid, etc. in water, resulted in the formation of a compact or barrier type oxide layer with the thicknesses of few nm.^61,66,70−73,75,76,87,88,93−95^

**Figure 5:**
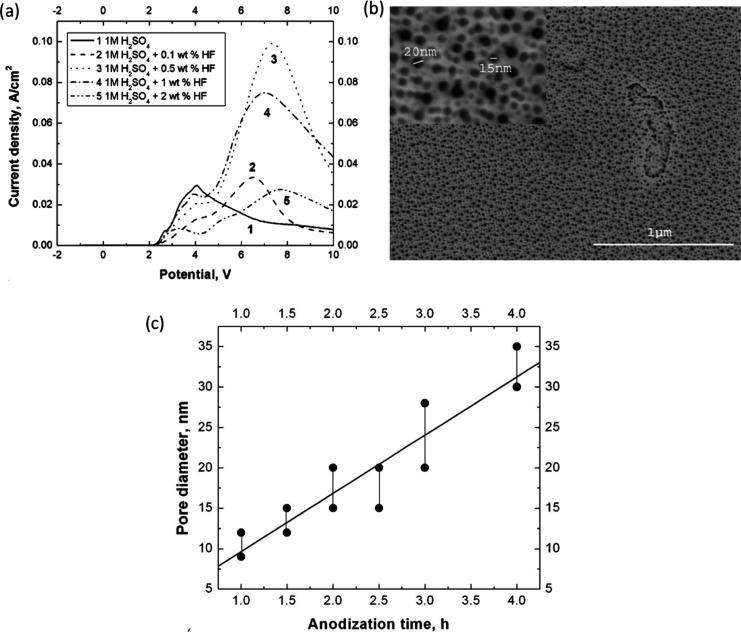
(a) Anodic polarization curves of tantalum in 1 M H_2_SO_4_ containing different concentrations of HF recorded at the scan rate of 10 mV s^–1^, (b) SEM image of the anodic porous tantalum oxide layer formed in 1 M H_2_SO_4_ + 2 wt % HF after 2 h of polarization at 20 V, (c) pore diameter of porous ATO layers formed in 1 M H_2_SO_4_ + 2 wt % HF for different anodization times. Reproduced with permission from ref (47). Copyright 2005 The Electrochemical Society.

The barrier ATO films are formed at both tantalum/oxide and oxide/electrolyte interfaces as a result of migration of O^2–^/OH^–^ and Ta^5+^ ions across the oxide layer, assisted by a high electric field.^93−95^ This compact structure is attributed to a very low solubility of anodic oxide layers in theses electrolytes. For example, Y. Kim et al. grew ATO films on Ta foil in citric acid solutions (0.01–0.2 M) at anodizing potentials up to 10 V and demonstrated that, for all the concentrations of citric acid, a compact ATO layer about 22 nm thick forms at 10 V.^102^ The incorporation of citrate anions in the ATO films was also evidenced from XPS analysis. This study further revealed that physical properties such as dielectric constant and interfacial capacitance values increase with the concentration of anodizing electrolyte (citric acid). M. V. Diamanti et al. showed a linear increase in thickness (45–180 nm) of ATO films with respect to preparation potentials (20–100 V) in either 0.5 M H_2_SO_4_ or 0.5 M H_3_PO_4_ electrolyte.^67^ In this study, it was demonstrated that the memristive behavior of the ATO films changes with film thickness. It is thoroughly investigated that barrier-type oxide films on tantalum are formed in almost all electrolytes, except for those containing hydrofluoric/fluoride ions. In the early 21^st^ century, substantial efforts were directed toward producing nanostructures of anodic Ta_2_O_5_ in electrolytes containing HF or NH_4_F.^43,53,92,96−98^ As previously mentioned, the evidence of the structural and morphological evolution of ATO nanostructures dates back to 2005 when H_2_SO_4_ containing a small amount of HF was employed as the anodizing electrolyte, with the anodizing potential intentionally kept well below the dielectric breakdown potential.^47^ A noticeable change in polarization behavior was observed in the presence of HF, which was characterized by higher current densities and the emergence of multiple broad peaks (Figure 5a). Furthermore, an increase in the concentration of HF up to a certain value (i.e., 1 and 2 wt %) resulted in a decrease in current densities, attributed to the formation of a new phase (TaF_5_) or changes in the morphology (i.e., pore formation) or effective surface area of the electrode. The formation of porous oxide layers and variations in the pore diameter with changes in the anodization time were observed for the first time (Figure 5b and c). Additionally, an increase in the thickness of the porous tantalum oxide layer with an increase in anodization time was observed. This study clearly indicated the role of HF in the formation of porous morphology and demonstrated that higher concentration (3 wt % or more) of HF can lead to partial or complete etching of porous oxide. This phenomenon may be attributed to an accelerated rate of oxide layer dissolution compared to the rate of oxide layer formation at the metal/electrolyte interface.

**Figure 6:**
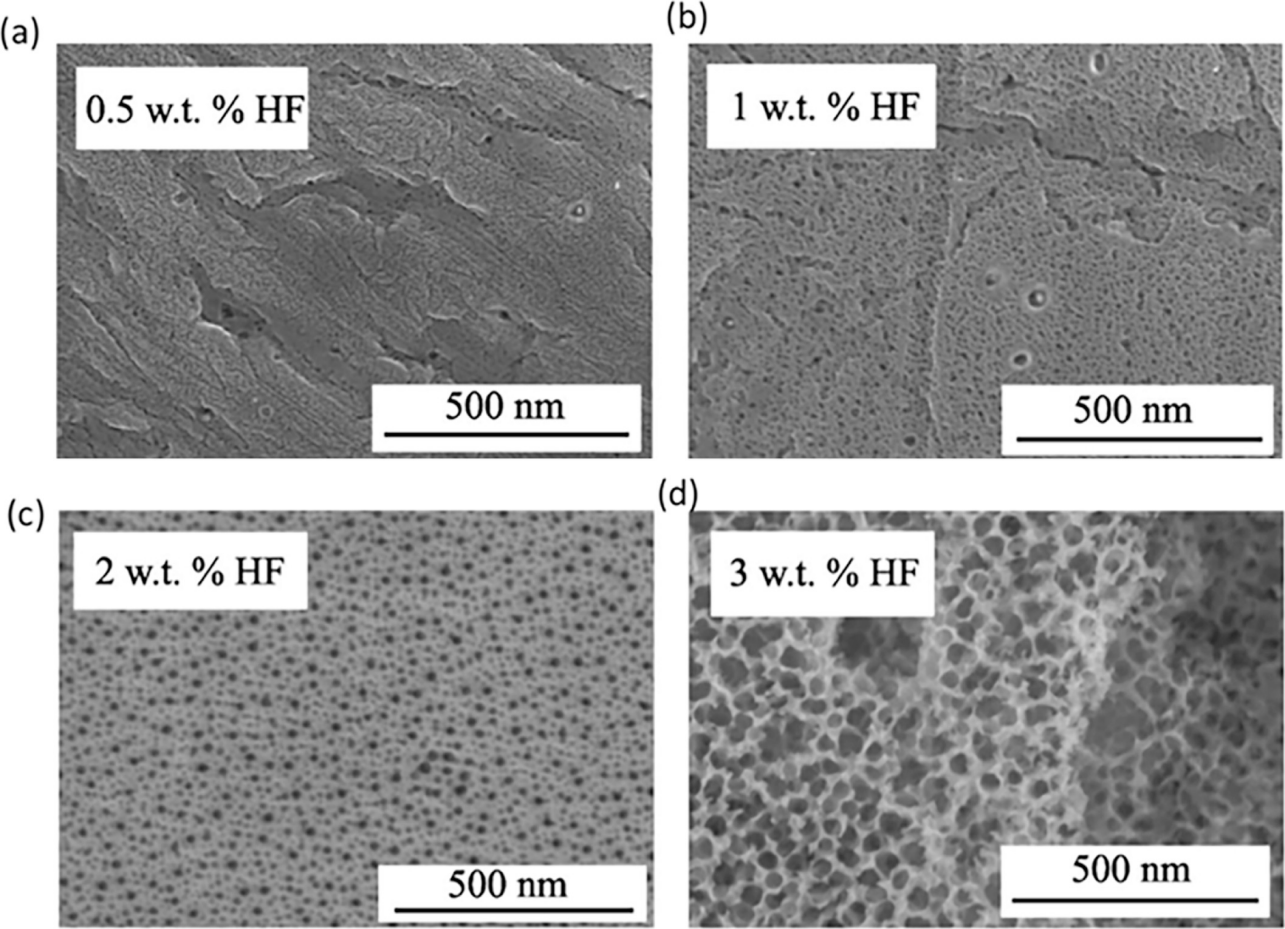
SEM images of the Ta surface anodized at 20 V (after a potential ramp from the *E*_OCP_ to 20 V with a scan rate 10 mV s^–1^ in H_2_SO_4_ (1 M) with different concentrations of HF (a) 0.5 wt %, (b) 1 wt %, (c) 2 wt %, and (d) 3 wt %. Reproduced with permission from ref (105). Copyright 2005 The Electrochemical Society.

**Figure 7:**
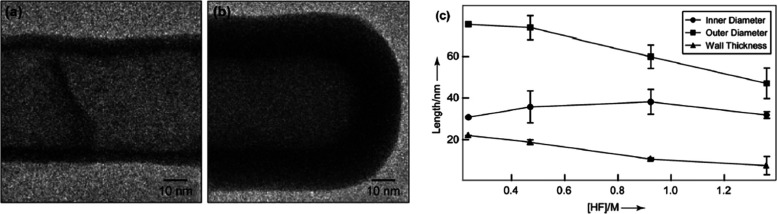
TEM images of (a) an open end of a Ta_2_O_5_ nanotube with thinner walls compared to (b) the other end of the same tube. (c) Graph showing a structural dependence of the inner diameter, outer diameter, and wall thickness on the HF concentration (4% H_2_O and 30 V). Reproduced with permission from ref (42). Copyright 2009 Royal Society of Chemistry.

Later on, the same research group undertook further optimization of the HF content in the electrolyte, aiming to produce porous tantalum oxide films with highly regular pore arrays featuring about 20 nm pore diameters.^43,105^ It was hypothesized that the interplay between pore formation and dissolution plays a critical role in the development of pores, order arrangement, and the thickness of the resulting oxide layer.^43,105−109^ SEM images of the materials prepared in this study are presented in Figure 6, which once again indicates a pivotal role of HF concentration in the generation of the nanoporous structure.^105^ This study also sheds light on the influence of other anodizing parameters on the oxide morphology, namely extending the duration of anodization (from 1 to 4 h) increases both the pore diameter (from 5 to 35 nm) and thickness (from 160 to 800 nm) of the porous ATO layer. Subsequent studies illustrated well-defined control over the morphology and dimensions of tantalum oxides by optimizing the ratio of electrolyte components, specifically HF:H_2_O:H_2_SO_4_. J. E. Barton et al.^42^ conducted an intensive study on the impact of the HF:H_2_O ratio and applied potential on the morphology of anodic Ta_2_O_5_ nanotubes encompassing such parameters as the inner diameter, outer diameter, and wall thickness (Figure 7).^42^ This study demonstrated that a high concentration of fluorine ions creates an optimal etching environment for the pore walls, resulting in nanostructures with larger diameters. However, it was noted that an excessive presence of fluoride ions in the electrolyte may lead to the production of disordered nanostructures due to permanent dissolution of the metal oxide. Apart from this, some studies reported the formation of nanoporous and nanotube morphologies of anodic Ta_2_O_5_ in a H_2_SO_4_ solution (1–16 M) containing a small amount of NH_4_F (0.8–3.3 wt %) as a fluoride ion source.^25,29^ All these results clearly indicate that various sources of fluoride ions can be chosen for the generation of ATO nanostructures. Pioneering work by C. A. Horwood et al.^54^ introduced a precise method for controlling the length of Ta_2_O_5_ nanotubes up to 50–1000 nm by manipulating the concentration of HF and anodizing time. They demonstrated the fabrication of uniform and short Ta_2_O_5_ nanotube (<100 nm) arrays by slowing down the rate of ATO nanotube growth through adjustment of HF concentration (0.2–1.1 M) in 16 M H_2_SO_4_. Notably, this study applied a relatively short anodization time, ranging from 2 s to 4 min at 15 V.

Similar to the anodization of Ti, it is presumed that the anodization of Ta in the presence of F^–^ may progress through the following steps.^29,53,91,106−109^ As mentioned earlier, when anodization starts, a compact oxide layer of TaO_x_ forms on the surface of tantalum.^29,53,91^ As the anodization progresses, the formation of pores occurs due to both phenomena, dissolution of oxide at the oxide/electrolyte interface and generation of stress at the metal/oxide interface. However, the first of these factors is more important in the initial formation of pores,^110,111^ and resulting Ta ions (Ta^5+^) appearing at the oxide/electrolyte interface, are soluble by forming [TaF_7_]^2–^.^29,109^ This process creates pores on the surface of the metal oxide films. It is hypothesized that the complex [TaF_7_]^2–^, having a lower charge-to-size ratio compared to the F^–^ ions, can easily be replaced by the electro-migrated F^–^ ions from the bulk solution.^91,105−109^ Thus, a new interface between oxide pores and electrolyte (specifically F^–^ ions) forms, and the above process repeats in a continuous manner during the entire length of anodization, potentially deepening the pores. The growth rate of nanoporous/nanotube structures may be determined by the diffusion rate of the [TaF_7_]^2–^ complex and F^–^ ions.^53,91^ It is predicted that the nanoporosity could be related to the molecular dimension of [TaF_7_]^2–^ and the formation kinetics of Ta_2_O_5_. A detailed analysis of various hypotheses for the formation of nanoporous/nanotube ATO structures and the proposed reaction mechanisms is discussed in Section 5 of this review.

**Figure 8:**
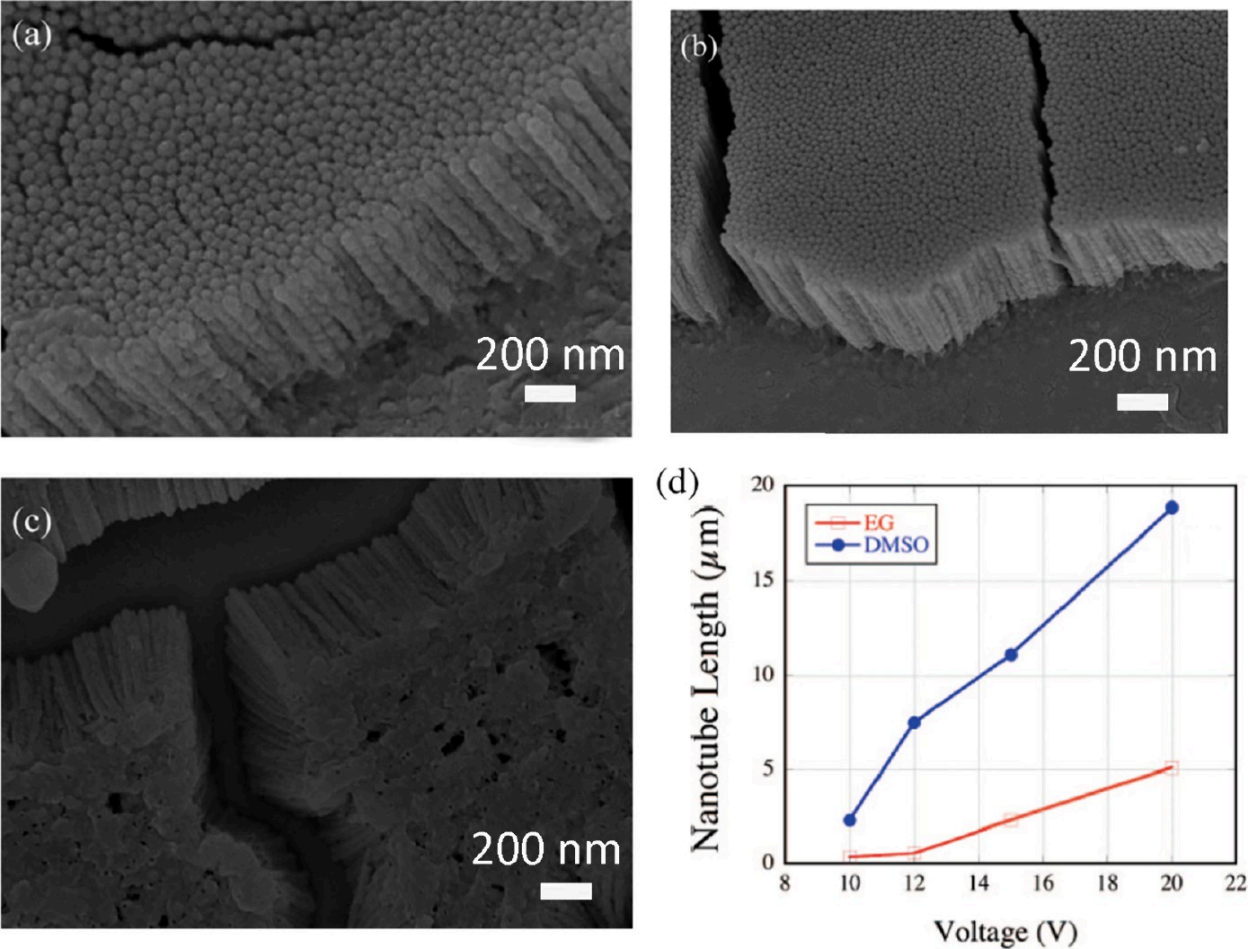
FESEM image of tantalum oxide surfaces formed in an electrolyte containing HF + H_2_SO_4_ (1:9 vol %) + 5 vol % ethylene glycol at (a) 10, (b) 12, and (c) 15 V. (d) Variation of the nanotube length as a function of anodization voltage for ATO samples fabricated in the presence of ethylene glycol (EG) and dimethyl sulfoxide (DMSO). Reproduced with permission from ref (57). Copyright 2008 American Chemical Society.

Further investigations into the variation of the composition of inorganic anodizing electrolytes include the addition of small quantities of organic solvents/electrolytes to the parent electrolyte or the creation of mixed inorganic–organic type electrolytes.^24,26,31,37,48,57^ N. K. Allam et al.^57^ demonstrated the fabrication of highly ordered nanoporous/nanotube ATOs in an H_2_SO_4_ + HF + H_2_O electrolyte containing low concentrations of other additives, such as ethylene glycol (EG), dimethyl sulfoxide (DMSO), or glycerol. They proposed that organic additives can alter various physiochemical parameters (e.g., viscosity, ion mobility, and current density) of the parent anodizing electrolyte and may play a crucial role in the development of the nanostructural morphology. Optimizing the anodization conditions, they demonstrated that the addition of 5 vol % ethylene glycol and the application of anodizing potentials of 10, 12, and 15 V are suitable for production of highly ordered ATO nanotubes (Figure 8a–c). They produced nanotube membranes up to ∼19 μm in length and demonstrated that the addition of DMSO resulted in the formation of nanotubes of larger length compared to those formed in EG containing solutions (Figure 8d).

**Figure 9:**
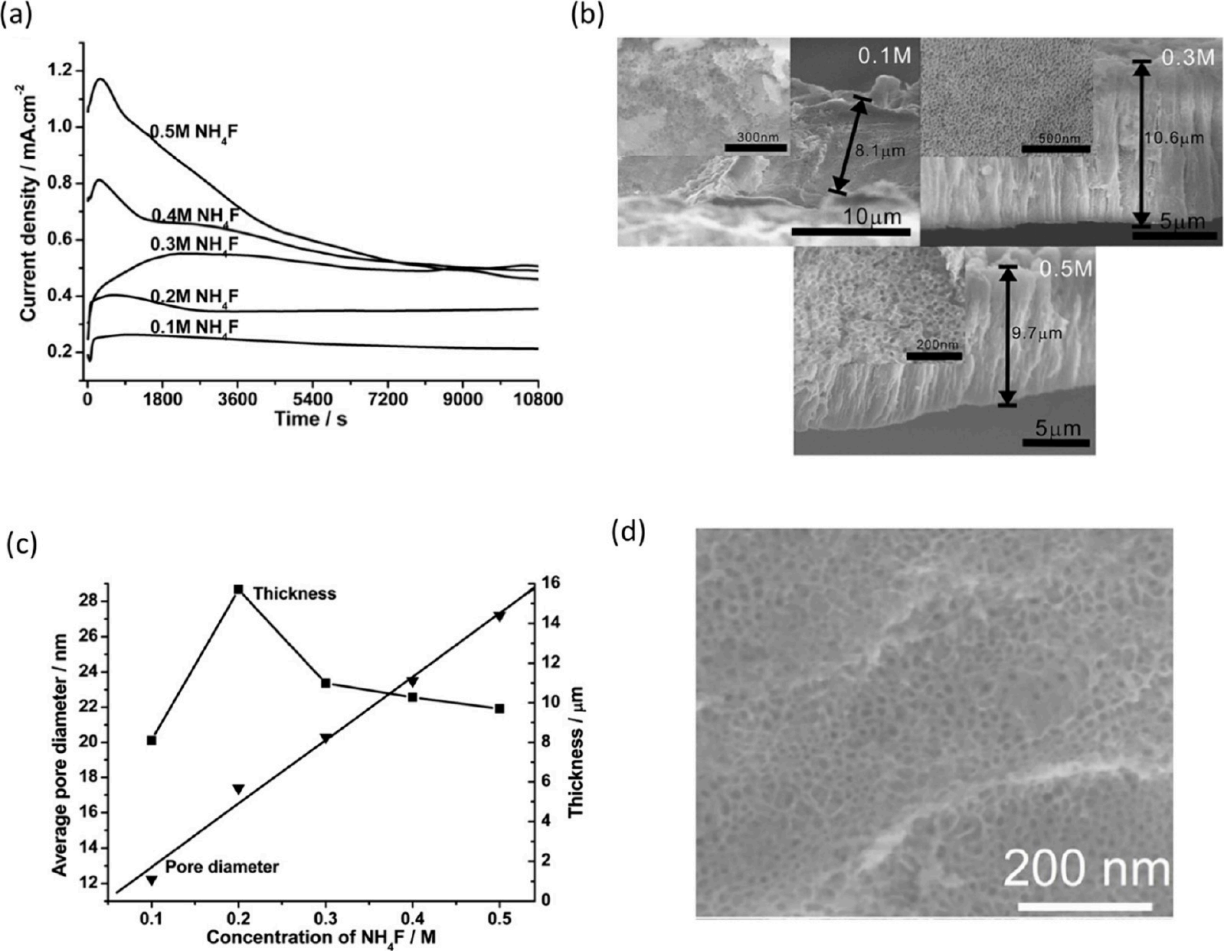
(a) Current density–time curves recorded during the anodization of Ta at 20 V for 3 h in glycerol with different amounts of NH_4_F. (b) SEM cross-sectional images of porous Ta_2_O_5_ layers grown as in (a) with 0.1, 0.3, and 0.5 M NH_4_F, the insets show corresponding top views of the anodic layers. (c) The average pore diameter (triangles) and the thickness (squares) of porous Ta_2_O_5_ layers prepared as in (a). Reproduced with permission from ref (62). Copyright 2008 Elsevier. (d) SEM image of top view of anodic Ta_2_O_5_ produced in EG with 0.2 M NH_4_F at 15 V for 2 h. Reproduced with permission from ref (40). Copyright 2018 Elsevier.

Similarly, nonaqueous anodization electrolytes, such as ethylene glycol (EG)/glycerol containing a small amount of NH_4_F, have been successfully formulated for the fabrication of ATO nanostructures.^37,40^ Moreover, numerous studies have further incorporated a small amount of H_2_O into the anodizing electrolyte containing EG/diethylene glycol (DEG)/glycerol + NH_4_F and examined the effect of water content on the morphology of anodic Ta_2_O_5_ layers.^21,26,44,62^ W. Wei et al.^62^ demonstrated the formation of nanoporous Ta_2_O_5_ layers by anodization of Ta in electrolytes composed of glycerol and NH_4_F (0.1–0.5 M). In this study, they highlighted the crucial role of the NH_4_F concentration and applied potential on the pore diameter and thickness of the anodic layers (Figure 9b, c). The study also revealed an increasing anodization current density with an increase in the concentration of NH_4_F in the electrolyte (Figure 9a). These results may suggest a higher growth rate of anodic structures in electrolytes containing higher NH_4_F concentrations. However, the volcano-shaped relationship between the concentration of NH_4_F and layer thickness suggests the possibility of dissolution of the anodic layer at high concentrations of NH_4_F (above 0.2 M). Similarly, for 0.2 M NH_4_F in EG as an electrolyte, S. Xia et al.^40^ observed a nanoporous ATO layer at anodizing potential of 15 V (Figure 9d). L. Fialho et al.^38^ achieved a nanoporous ATO layer by anodizing Ta foil in EG containing 3 vol % H_2_O and NH_4_F (0.3–2.4 wt %) at 40 and 60 V. They demonstrated that under different concentrations of NH_4_F (1.2 and 2.4 wt %) in the electrolyte, an increase in the applied electric field (60 V) could generate uniform pores on the surface layer. However, the precise role of NH_4_F in the formation of ATO layers was not clearly indicated in this study.

Additionally, some studies demonstrated the effect of electrolyte temperature on the nanoporous/nanotube morphology of ATO films.^27,48^ R. V. Gonçalves et al. systematically investigated the change in ATO NT length and pore diameter with variation in electrolyte temperature.^27^ In H_2_SO_4_ + 1 vol % HF + 4 vol % H_2_O electrolytes at an anodizing potential of 50 V for 20 min, it was demonstrated that the length of NTs increased gradually from 1.3 to 4.6 μm with an increase in electrolyte temperature from 0–50 °C.^27^ This study further revealed a gradual decrease in the diameter of NTs from 143 to 90 nm with an increase in the electrolyte temperature. K. Lee et al. showed the relationship between anodizing potential and electrolyte temperature for the formation of highly ordered nanoporous ATO films.^48^ For 1 h of anodization at 20 V (10 wt % K_2_HPO_4_ containing anhydrous glycerol), it is demonstrated that an electrolyte temperature of 180 °C is suitable for the production of ordered nanoporous ATO films with a pore diameter of ∼25 nm. It is further illustrated that higher (200 °C) or lower (150 °C) electrolyte temperatures may promote irregularity in the pores.

### 4.4 Effect of F^–^ and Water Content

As discussed in the earlier sections, an appropriate concentration of fluoride ions in both aqueous and nonaqueous electrolytes is crucial to create a suitable environment for the formation of nanoporous/nanotubular ATO layers.^21,26,43,44,53,92,96−98^ However, the influence of H_2_O addition to nonaqueous electrolytes or the variation of the F^–^ ions to H_2_O ratio in both aqueous and nonaqueous electrolytes can prompt a new growth model for ATO structures and therefore needs separate discussion.^49,58,62^

**Figure 10:**
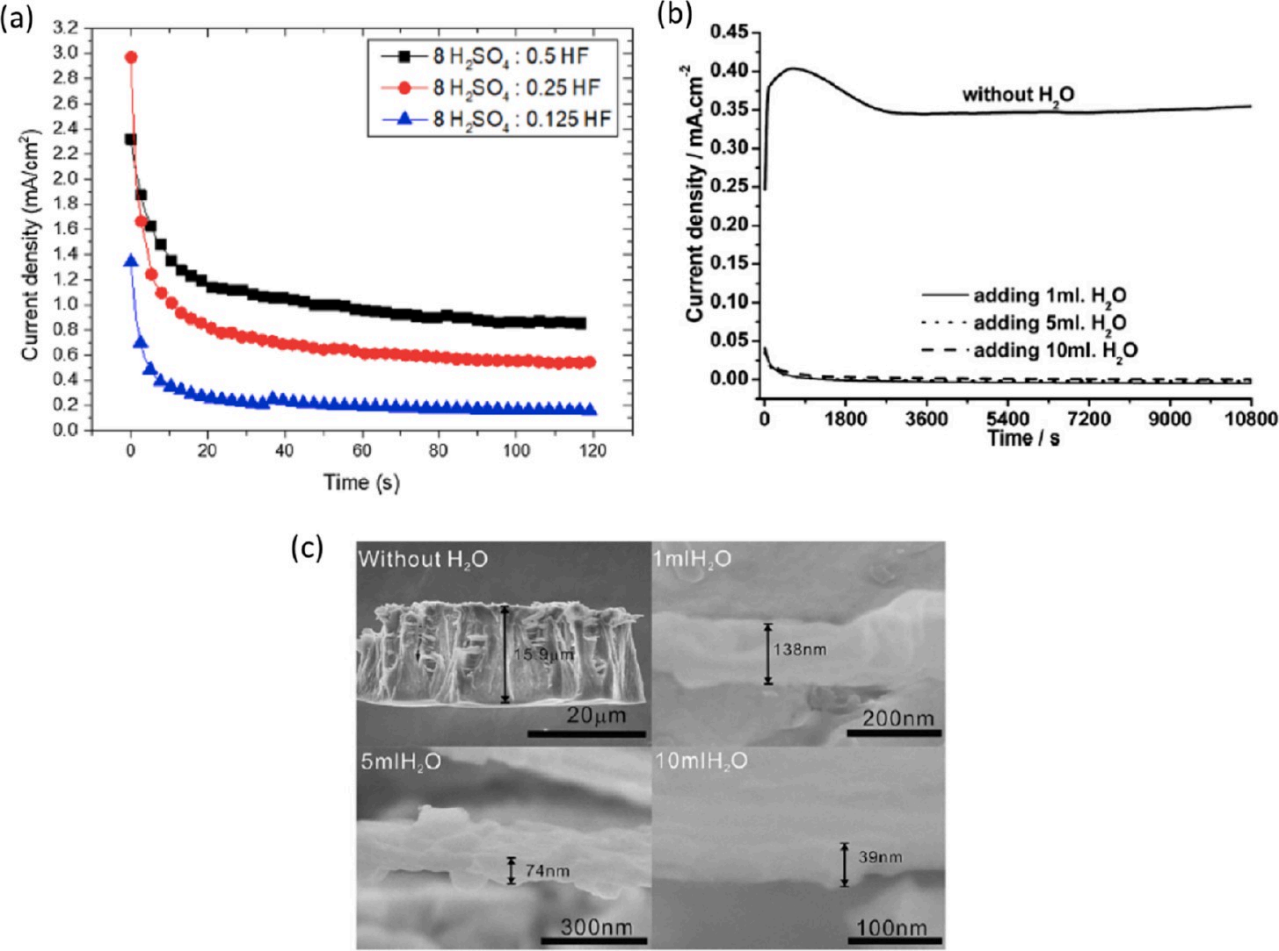
Current density vs time curves for Ta anodization in (a) H_2_SO_4_ (8 vol %) with different concentrations HF (from 0.5 to 0.125 vol %) at room temperature under 15 V for 120 s. Reproduced with permission from ref (49). Copyright 2020 Elsevier. (b) in glycerol +0.2 M NH_4_F electrolyte containing different amounts of water at 20 V for 3 h, and (c) SEM cross-sectional images of Ta_2_O_5_ layers grown at the conditions indicated in (b). Reproduced with permission from ref (62). Copyright 2008 Elsevier.

In the case of concentrated mixtures of H_2_SO_4_ (8 vol %) + HF (0.125–0.5 vol %), it has been observed that the anodization current density decreases as the HF concentration decreases to 0.125 vol % (Figure 10a).^49^ It has been previously noted that F^–^ ions can attack and dissolve the anodic oxide layer through pitting corrosion. Therefore, the decrease in the HF concentration can be associated with a decrease in the dissolution rate of the ATO layer. Consequently, a decrease in the F^–^ concentration and an increase in the H_2_O content can help stabilize the anodic oxide by decreasing the dissolution rate. Therefore, nanostructures (nanoporous/nanotubular) of ATO cannot form on the initial compact oxide layers unless the H_2_O/F^–^ ion ratio is adequately balanced in the electrolyte.

M. A. A. Talip et al. and W. Wei et al.^58,62^ systematically explored the influence of the H_2_O content on the morphology of ATO layers. It is observed that the addition of H_2_O (1–5 mL) to 200 mL of glycerol +0.2 M NH_4_F resulted in a rapid decay of the anodizing current density from the initial value (Figure 10b). SEM image studies demonstrated that the addition of water to glycerol + NH_4_F-based electrolytes promotes the formation of a compact or inhomogeneous porous oxide layer (Figure 10c). M. A. A. Talip et al.^58^ varied the H_2_O content (0, 2, 4, and 6 vol %) in the anodizing electrolyte (EG containing 1.35 wt % NH_4_F and 0.25 vol % of H_2_SO_4_) and demonstrated that no nanostructures could be produced without H_2_O. In contrast, nanotubular structures could be formed in the electrolyte containing 2 and 4 vol % of H_2_O. Hence, these studies confirmed that an optimum amount of H_2_O in both aqueous and nonaqueous electrolytes may help promote the nanostructural ATO morphology by manipulating the dissolution rate.

### 4.5 Effect of Anodization Potential, Current Density, and Time

As discussed in earlier sections, apart from the electrolyte composition, anodization parameters, such as anodization potential/current density and duration of anodization, can also contribute to the morphological evolution of anodic ATO layers.^13−18,28^ M. Fernández demonstrated a linear increase in voltage during a constant current (0.04 A) anodization of the Ta electrode in 0.01–10% H_3_PO_4_ or 0.01 H_3_PO_4_ + 0.1–0.15% citric acid solutions.^94^ They observed a gradual increment in voltage over time until an oscillation appeared in the voltage range of 100–200 V. The oscillations in the *V* vs *t* curve indicated breakdown events, and the voltage at which the oscillations start is known as the scintillation voltage. These oscillations suggest poor electrical conductivity or high resistivity of the oxide layer. Many earlier studies on Ta anodization demonstrated minimal effects of anodization potential/current and time on the morphology of anodic tantalum oxide layer when anodized in one component electrolytes, such as Na_2_SO_4_, H_2_SO_4_, H_3_PO_4_, chromic acid, citric acid etc.^41,52,61,63,66,70−73,75,76,79,87−89,94−96,101^ However, studies in the early 21^st^ century revealed a profound effect of anodization potential/current and time on the morphology of anodic tantalum oxide layer when formed in fluoride-containing electrolytes.^33 −40 ,43 ,53 ,92 ,96 −98^ In this section, we summarize some notable investigations in these directions.

**Figure 11:**
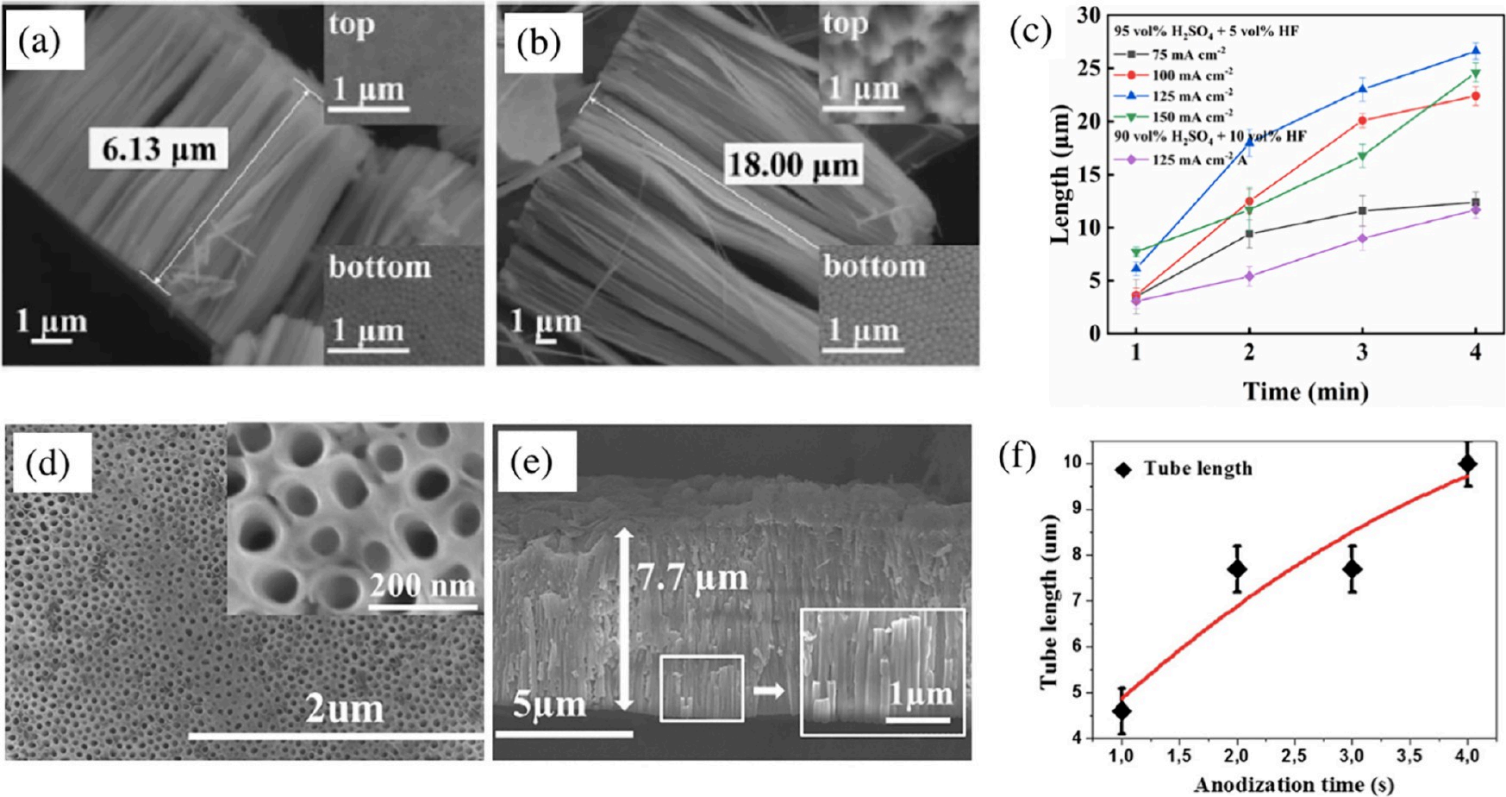
SEM images of the cross-sectional views of Ta_2_O_5_ nanotubes obtained in the electrolyte containing 95 vol % H_2_SO_4_ + 5 vol % HF at 125 mA cm^–2^ for different anodizing times: (a) 1 min and (b) 2 min. (c) The length of Ta_2_O_5_ nanotubes as a function of anodizing time in the electrolyte containing 90–95 vol % H_2_SO_4_ + 5–10 vol % HF at different current densities. Reproduced with permission from ref (74). Copyright 2021 Elsevier. (d) Top and (e) cross section SEM images of Ta_2_O_5_ nanotube arrays grown in sulfuric acid–based electrolyte containing 13.6 wt % H_2_O and 0.8 wt % NH_4_F under galvanostatic anodization at 1.2 A cm^–2^ for 2 s. (f) corresponding the layer thickness vs anodization time curve. Reproduced with permission from ref (25). Copyright 2015 Elsevier.

T. Wen et al.^74^ conducted a study on the anodic growth of Ta_2_O_5_ nanotubes under the constant anodization current (75–175 mA cm^–2^) in a H_2_SO_4_ (90–95 vol %) + HF (5–10 vol %) electrolyte. This research revealed an increase in the length and diameter of nanotubes with an increase in the anodizing current up to 150 mA cm^–2^. It was specifically observed that only the length of nanotubes is directly proportional to the anodizing time and the growth rate of nanotubes decreases with an increase in time or HF concentration. A growth rate of 9.0 μm min^–1^ was observed for the first 2 min at 125 mA cm^–2^ in an electrolyte containing 5 vol % HF (Figure 11a–c). In another work, Z. Su et al.^25^ demonstrated constant current anodization at 1.2 A cm^–2^ with an unprecedently high growth rate of Ta_2_O_5_ nanotubes, reaching the length of ∼7 μm in just 2 s and ∼10 μm in just 4 s. In this case, H_2_SO_4_ with 13.6 wt % H_2_O and 0.8 wt % NH_4_F was chosen as the anodizing electrolyte (Figure 11d–f).

**Figure 12:**
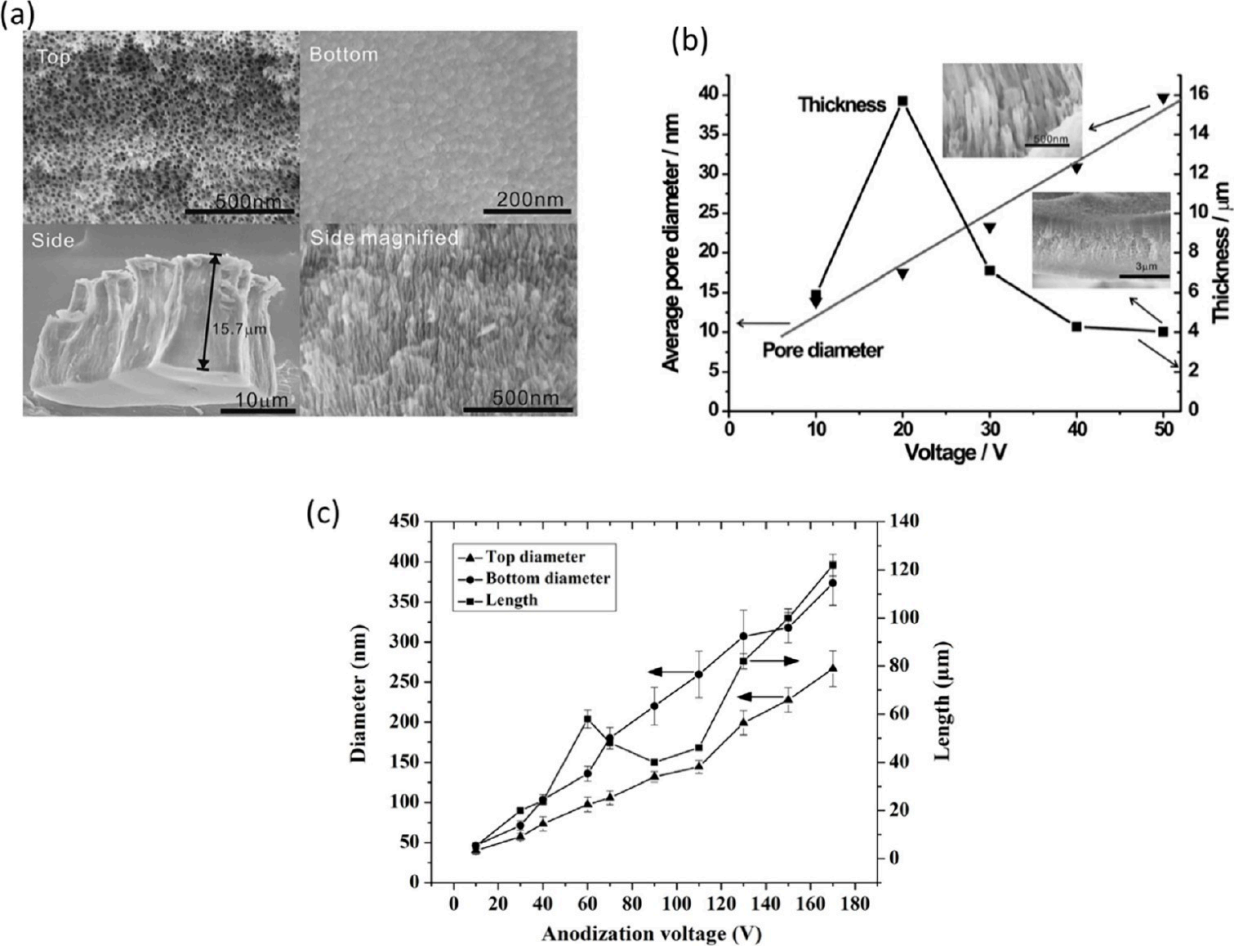
(a) SEM images of the anodic nanoporous Ta_2_O_5_ film formed in glycerol +0.2 M NH_4_F electrolyte during 3 h-anodization at 20 V showing top-, bottom-, cross-sectional, and a magnified cross-sectional view. (b) The average pore diameter (triangles) and the thickness (squares) of porous Ta_2_O_5_ layers prepared in glycerol +0.2 M NH_4_F electrolyte for 3 h at different potentials ranging from 10 to 50 V, insets show SEM images of the nanoporous Ta_2_O_5_ layer grown at 50 V. Reproduced with permission from ref (62) Copyright 2008 Elsevier. (c) Variation of the nanotube diameter (top diameter and bottom diameter) and nanotube length as a function of anodization voltage (10–170 V) for samples fabricated after 1 h anodization. Reproduced with permission from ref (45) Copyright 2017 Elsevier.

Similar to the anodizing current, the anodizing voltage also plays an essential role in the evolution of the nanostructural morphology of anodic ATO (Figure 12a). W. Wei et al.^62^ observed a volcano-shaped relationship between the thickness of the porous ATO layers and the applied potential (10–50 V) in glycerol containing 0.2 M NH_4_F (anodization time of 3 h). Initially, the thickness of the porous oxide film increases steeply from about 5.9 μm at 10 V to 15.7 μm at 20 V and then decreases with an increase in the anodizing potential beyond 20 V (Figure 12b). In this study, the pore diameter of the ATOs was demonstrated to depend linearly on the applied potential. Hence, it was predicted that high anodizing voltage (above 20 V) can speed up the dissolution or etching process, which can directly affect the thickness of the layer and pore diameter of ATO. Moreover, anodization voltage can also regulate the interpore distances in the nanopore/nanotube ATO films.^42,49,109^ In H_2_SO_4_ containing 0.25 vol % HF and 4.3 vol % H_2_O, J. E. Barton et al.^42^ demonstrated that the spacing between ATO nanotubes is directly proportional to the anodization voltage, with a proportionality constant of nearly 0.0025 μm/V for the applied voltage range of 10 to 90 V. This study further demonstrated that the NTs produced at 10 V and at higher voltages (40–90 V) were disordered with variations in pore diameter along the nanotubes.^42^ For example, the outer diameters varied from ∼30 nm at the ends to ∼20 nm in the middle for the NTs produced at 10 V. W. Chen et al.^45^ fabricated three types of ATO films (free-standing ATO nanotube membranes, highly ordered amorphous adhered ATO nanotube arrays, and spark discharge films of ATO crystal phase) in the same electrolyte composed of concentrated H_2_SO_4_ and HF. This study further confirmed an increase in the diameter of ATO nanotubes with an increase in anodization voltage (Figure 12c). L. Fialho et al.^38^ synthesized nanoporous ATO layers at 40 and 60 V in ethylene glycol containing 3 vol % H_2_O and 0.6–2.4 wt % NH_4_F. They observed randomly ordered porous layers of ATOs at both anodizing potentials. It is assumed that an imbalance between the rate of oxide formation and dissolution is the reason behind the formation of disordered porous structures.

However, the debate on conditions for the separation of nanopores and its transformation to nanotubular ATO films can be clearly understood from the work of C. F. A. Alves et al.^49^ As demonstrated in Section 4.2, the EDX elemental mapping of the single free-standing ATO NT showed the presence of S and F with a high F/S atomic ratio (1.8) on the NT wall (Figure 4). From this, it is hypothesized that F^–^ ions, having a higher migration velocity compared to that of O^2–^, can migrate from the outer part of the oxide layer to the inner part, creating a wall of separation between pores and thus being responsible for the separation of pores. Thereafter, the degree of solubility of the [TaF_7_]^2–^ complexes present on these walls, and their diffusion in an electrolyte containing optimized amount of H_2_O, may influence the nanotube morphology. This study demonstrated the formation of free-standing ATO NTs with a diameter of ∼45 nm at 15 V in a H_2_SO_4_ electrolyte containing 0.5:1.5 vol % of HF:H_2_O. Moreover, efforts have also been made to understand the effect of anodizing voltage and time on ATO structures in fluoride free electrolytes. K. Lee et al.^48^ synthesized porous ATOs in a F^–^ ion free nonaqueous electrolyte containing glycerol and 10 wt % K_2_HPO_4_ at 180 °C. This study showed the formation of highly ordered self-organized porous structures (pore size of ∼25 nm) at the anodization potential of 20 V and irregular porous structures at 2 and 50 V. However, the reason behind the observed anomaly in the morphology with the change in applied potential was not properly described in this study. From the above discussions, it can be concluded that the anodizing voltage/current and time are equally important parameters as the composition of the electrolyte. We anticipate plenty of opportunities to generate new kind nano/micro structures of ATOs simply by controlling these parameters. More details on the effect of anodizing potential/current density and time on the morphology can be found in Table 4.

### 4.6 Problems with Adhesion of Anodic Layers to Substrates

The synthesized ATO layers can undergo further processing depending on their utility or the mode of application. Generally, a well attached anodic oxide layer to the Ta substrate is preferred for photo or photoelectrochemical reactions.^21,23−25,86^ However, numerous studies have showed weak adhesion of ATO layers to the substrate, reporting that these layers can be detached completely or partially during washing in water.^27,28,53,66,72,93^ This poses a significant challenge for their direct application in many sectors. It is evident that very long NTs of few μm in length carrying high mass pose a risk of detachment from the metal substrate.

**Figure 13:**
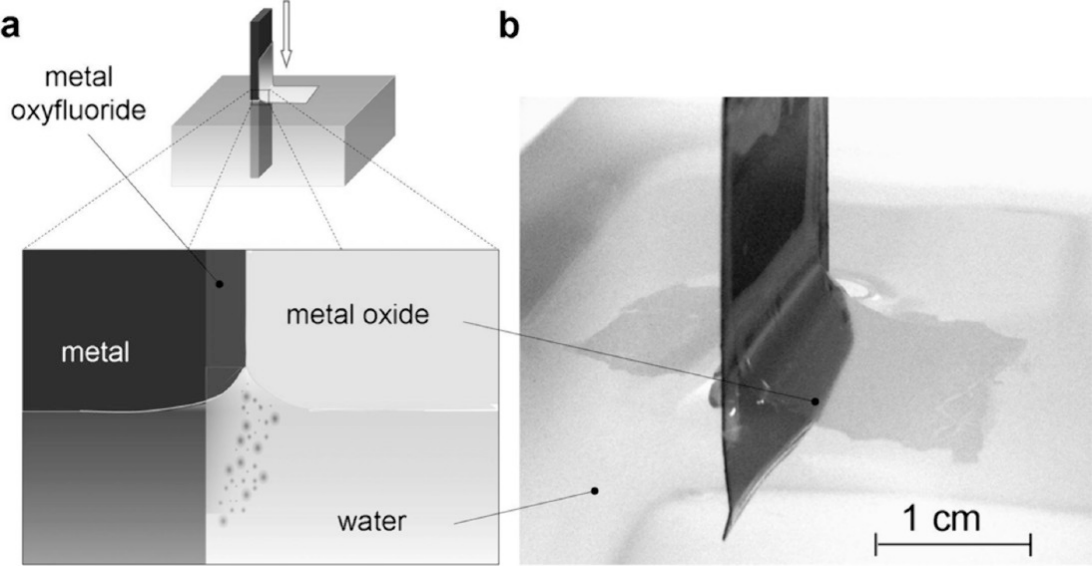
(a) Pictorial demonstration of the film peeling process. The architecture of the oxide film composition is shown as separated layers. The film detaches due to the dissolution of the fluoride-rich layer in water and floats off due to the skin effect. (b) Micrograph of a partially detached and floating tantalum oxide film over the water surface. Reproduced with permission from ref (97). Copyright 2008 American Chemical Society.

Many studies have indicated that the incorporation of fluoride ions in the anodic films is the major reason behind the detachment of the film.^27,28,53,92,97^ As discussed earlier, the inward mobility of fluoride ions is almost twice to that of O^2–^ ions, leading to the formation of a thin layer of fluoride and/or oxyfluoride species (TaF_5_, TaO_2_F and TaOF_3_) between the anodic oxide film and tantalum substrate.^97^ Alternatively, it can be said that a gradual build-up process of fluoride matrix at the Ta/Ta_2_O_5_ interface occurs throughout the anodization process.^53,57,92^ These species are predicted to be fairly soluble in water and can therefore be easily detached by washing or during ultrasonication treatments (Figure 13). Some studies have even reported that ATO films can be detached completely with minimal handling.^53,66,72,92^ It is noteworthy that in the case of other anodic structures, such as TiO_2_ and WO_3_, the films are difficult to detach due to differences in the solubility of the oxyfluoride–metal complexes for these samples.^97^

However, in the previous decade, many efforts were made to synthesize ATO NTs that are well adhered to the Ta substrate.^27,51^ M. A. Baluk et al.^51^ studied the crucial role of anodization potential, time and post annealing effects on the adhesion properties of ATO NTs prepared in F^–^ ion-containing H_2_SO_4_ solutions. It was demonstrated that ATO NTs (length of 1.68–2.19 μm) formed at 15 V for 5 min were uniform and strongly adhered to the Ta substrate. Moreover, the morphology and adhesion of the NTs seemed to be unaffected at an annealing temperature up to 450 °C in a nitrogen atmosphere. On the other hand, NTs produced at 20 V for 10 min possessed weak adhesion properties and can be easily detached from the surface. Most importantly, they demonstrated that an annealing temperature of 600–700 °C can defragment the NT arrays and the base Ta foil. R. V. Gonçalves et al.^27^ explored the crucial role of electrolyte temperature on the adhesion of Ta_2_O_5_ NTs. They systematically investigated the effect of electrolyte (H_2_SO_4_ + 1 vol % HF + 4 vol % H_2_O) temperature on the adherence properties of ATO NTs and demonstrated that ATO NTs produced at 0, 10, and 20 °C were well adhered to the substrate. Meanwhile, the ATO NTs produced at above 30 °C started to detach from the Ta substrate or could be easily removed by an adhesive tape.

## 5 Various Hypotheses Linked to Anodization and Anodization of Tantalum

In general, the morphology of anodic tantalum oxide layers can be categorized into four types: (a) a compact layer with a microstructural morphology, (b) nanodimple, (c) disordered porous, and (d) ordered nanoporous/nanotubular structure.^21−105^ As discussed earlier, anodizing conditions like appropriate F^–^ ions containing electrolytes, voltage range, and anodizing time are necessary to shape the metal oxide layer into a nanotube/nanopore geometry, and this can be correlated to an equilibrium condition between the oxide formation and its dissolution. In this section, we will discuss all possible growth mechanisms of ATO in electrolytes containing F^–^ ions or free of F^–^ ions.

### 5.1 Field-Assisted Model for the Porous and Tubular ATO Growth

**Figure 14:**
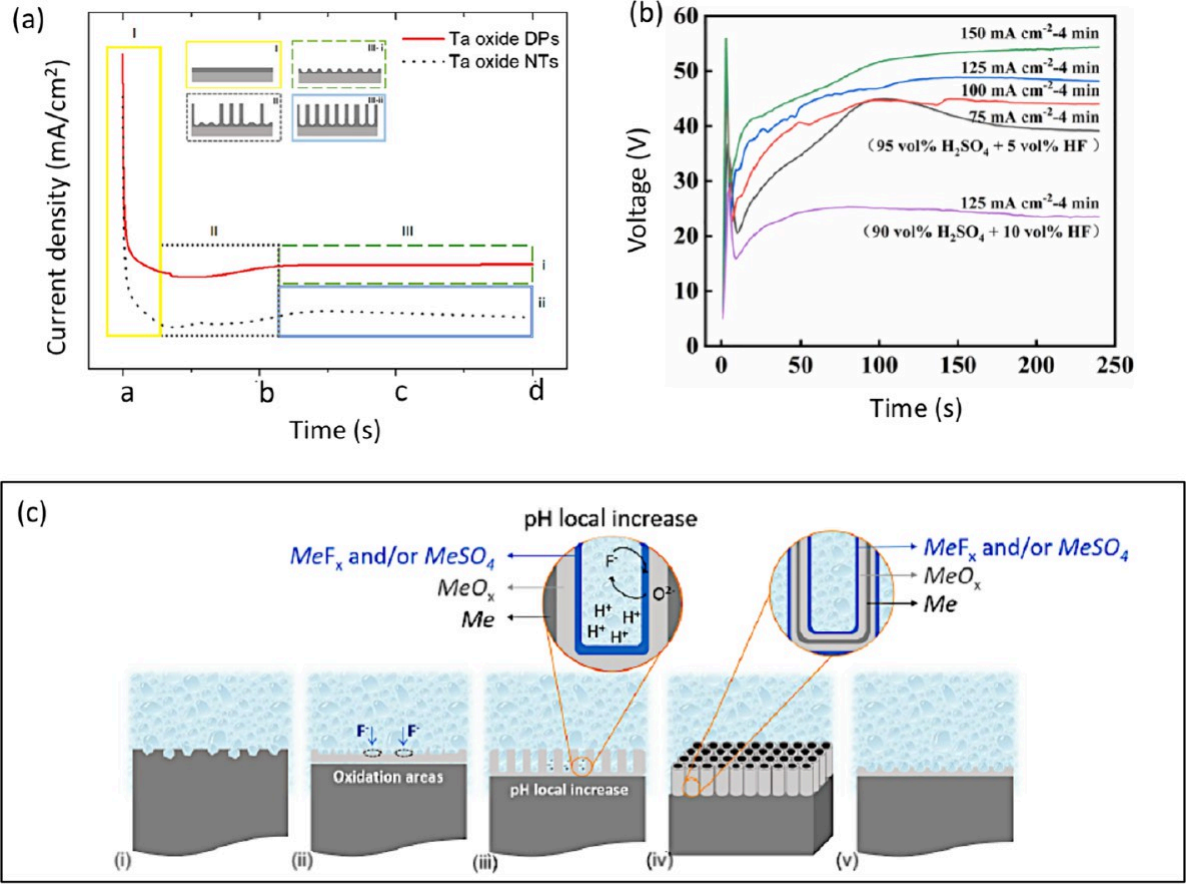
(a) A visual representation of current density-time response during Ta anodization with the typical three stages: (I) a compact oxide layer formation, (II) pore formation and extension, and III) steady-state growth with (i) dimples (DPs) formation or (ii) nanotubes (NTs) formation. Reproduced with permission from ref (38). Copyright 2020 Elsevier. (b) Voltage–time curves recorded during constant current anodization in the electrolyte containing 90–95 vol % H_2_SO_4_ + 5–10 vol % HF at different anodizing current densities. Reproduced with permission from ref (74). Copyright 2021 Elsevier. (c) Schematic representation of the nanopore-nanotube assembly mechanism: (i) a surface with pits before anodization; (ii) dissolution of the Ta_2_O_5_ anodic layer and formation of dimples; (iii) growth of nanopores (length grown by electric field); (iv) formation of nanotubes; (v) dimple-shaped morphology. Reproduced with permission from ref (49). Copyright 2020 Elsevier.

The most important information regarding the growth mechanism of tantalum oxide can be understood from the current–time (*i*–*t*) plot for the constant potential anodization, and from the potential-time plot for galvanostatic anodization (Figure 14a,b).^38,49,74^ Typically, three different stages of the ATO growth can be identified in the *i*–*t* curve recorded during anodization in an electrolyte containing fluoride ions (Figure 14a).^38^ An exponential decay in current/voltage at stage (i) is proposed to be associated with the formation of a resistive compact oxide layer through Ta hydrolysis at the metal/electrolyte interface (Figure 14a, c, stage I). This phenomenon is expressed in eq 1. Thereafter, the anodic current reaches a minimum value, believed to be the onset of electric-field-enhanced dissolution of the oxide film through fluoride ion etching. These processes may lead to the initiation of small pores on the oxide layer. From this point onward, ions present in the electrolyte (OH^–^ and F^–^) and in the oxide layer (Ta^5+^ and O^2–^) can interact and move through the barrier-type oxide layer. This results in the continuation of the oxidation process with an increase in the anodic current. At this stage, porosity is thought to be induced by the acidic environment containing F^–^ ions (Figure 14a and c, stage II). As the pores extend, the surface area consecutively increases, potentially enhancing the rate of ATO dissolution (eq 2). Finally, a steady state of the anodization/oxidation current can be maintained thereafter (Figure 14a, stage III). This may indicate an equilibrium between the oxide formation and dissolution reactions:
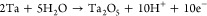
1
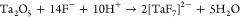
2

According to L. Fialho et al.,^38^ there are two possibilities in the third stage. For the first one, a decrease in the anodization current density over time is observed. This can result in the formation of stable nanotube/pore structures (Figure 14a, stage III-ii). Alternatively, a steady-state anodic current or a very slow increase in the current may occur. This observation indicates instability in the nanopore/tube structures, resulting in a dimpled surface morphology (Figure 14a, stage III-i).

**Figure 15:**
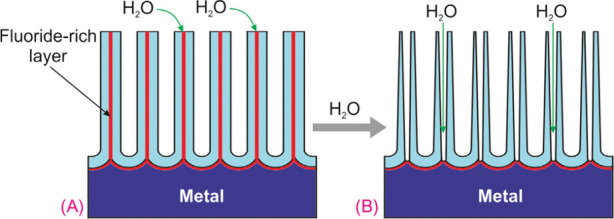
Schematic representation of nanotube formation from nanopores by a selective dissolution of the fluoride-rich layer by H_2_O molecules from the electrolyte. (A) Nanoporous anodic oxide with the fluoride-rich layer at cell boundaries and pore bottoms. (B) Anodic oxide nanotubes formed by selective etching of the fluoride-rich layer. Reproduced with permission from ref (15). Copyright 2020 Elsevier.

Spectroscopic evidence supporting the growth mechanism of ATO structures aligned with the *i*–*t* anodization curve can be found in the works by Alves et al. and M. A. A. Talip et al.^49,59^ These researchers put forth a plausible explanation for the growth of ATOs in the H_2_SO_4_ + HF electrolyte by analyzing HAADF-STEM images and STEM-EDS compositional maps of the dimple and pore structures. Their proposal involves a chemical dissolution of Ta_2_O_5_ at the oxides/electrolyte interface, which is enriched with F^–^ ions, by fluoride/sulfate ions. It is considered that a small ionic radius of F^–^ allows to penetrate the growing Ta_2_O_5_ lattice and thus be transported through the oxide layer by the applied field. In accordance with eq 1, the concentration of H^+^ ions may increase in the vicinity of the oxide layer with a nanodimple morphology (Figure 14c, (ii). To maintain electroneutrality, F^–^ ions are hypothesized to migrate to the H^+^ sites, potentially competing for the position of the O^2–^ in the oxide lattice. Subsequently, the dissolution of Ta_2_O_5_ is initiated through the formation of the [TaF_7_]^2–^ complex, as described by eq 2, leading to the creation of the porous layer structure (Figure 14c, (iii). The dissolution of Ta generates negatively charged cation vacancies at the oxide surface, potentially enhancing the reaction described in eq 1. Consequently, Ta^5+^ ions can easily migrate to nearby vacancy sites, leading to deeper pore formation. The transition from a porous to a tubular structure can be visually represented in Figure 14c, (iii), and Figure 15. The fluoride-rich layer that separates the pores is susceptible to dissolution in water, giving rise to a nanotubular morphology.^15,49,59^ Therefore, the interplay between the rate of electric field-assisted metal oxide formation in a fluoride containing electrolyte and its chemical etching favors the development of the nanoporous/nanotubular structure. Moreover, it is proposed that the bottom of the pores/tubes are more acidified compared to their top.^49,15^ As a result, the rate of oxide dissolution at the bottom of pores/tubes is higher compared to that of pores/tubes at the top, which contributes to an increase in the length of the pore/tubular structure. Consequently, it can be inferred that the length and diameter of nanopores/nanotubes are dependent on parameters such as the concentration of fluoride ions, applied potential/current density, and the duration of the process.

## 6 Post-treatment of Anodic Films and Their Characterization

**Figure 16:**
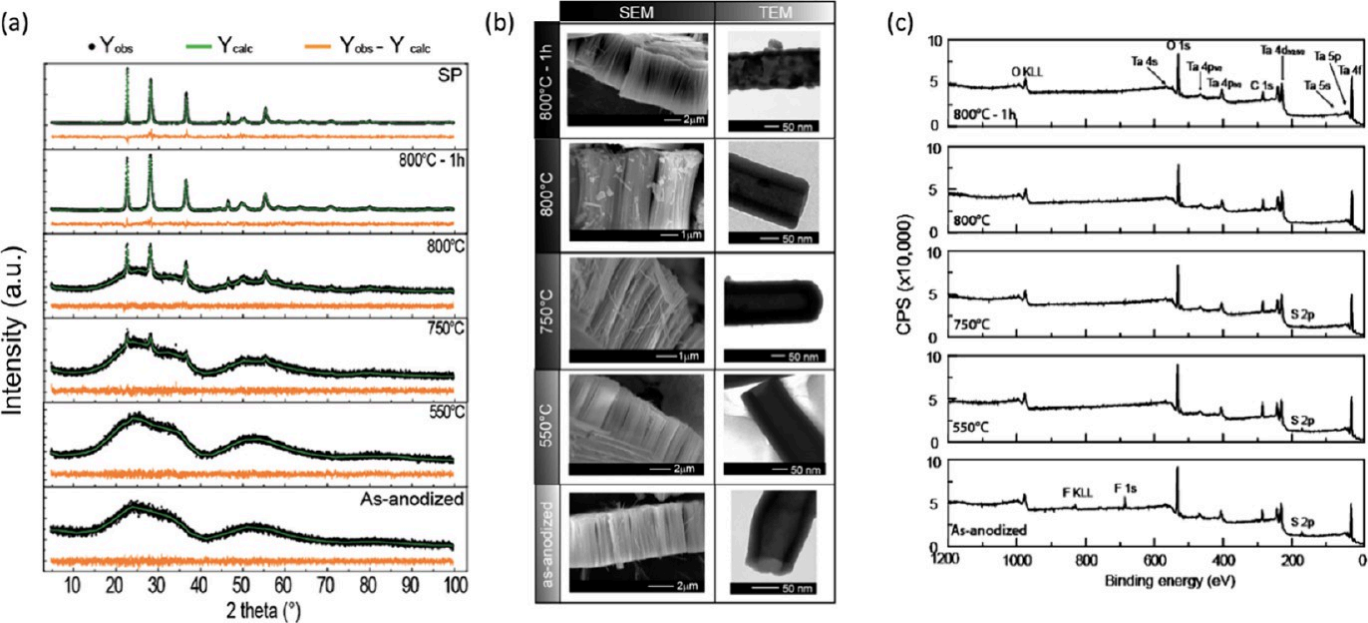
(a) XRD diffractograms of as-anodized and annealed freestanding Ta_2_O_5_ NTs. NTs were prepared at 50 V in an electrolyte (H_2_SO_4_ + 1 vol % HF + 4 vol %) at 50 °C and then annealed at 550, 750, 800 °C for 0.5 h and at 800 °C for 1 h. The top diffractogram was recorded for the commercial Ta_2_O_5_ powder sample (SP). (b) SEM and TEM images of the freestanding Ta_2_O_5_ NTs prepared at a potential of 50 V under the same anodization conditions as in (a). All samples were annealed at different temperatures for 0.5 h, except the bottom image that shows Ta_2_O_5_ annealed for 1 h at 800 °C. (c) Survey XPS data of the freestanding Ta_2_O_5_ NTs prepared at a potential of 50 V with the anodization electrolyte at 50 °C. All samples were annealed at different temperatures for 0.5 h, except the top spectrum that shows Ta_2_O_5_ annealed for 1 h at 800 °C. Reproduced with permission from ref (27). Copyright 2012 American Chemical Society.

The freshly prepared anodic tantalum oxide films on Ta are often amorphous. Therefore, when examining the freshly prepared ATOs, the XRD pattern of the Ta substrate (JCPDS, No. 89–5158) is observed.^112,113^ Additionally, as discussed in earlier sections, the anodic structures may contain a significant amount of impurities from the electrolytes, e.g., F, S, P, etc.^36−39,69,75^ Numerous studies have demonstrated that thermal treatment plays an important role in the elimination of the impurity content, and enhances the crystallinity of the nanostructures, which can ultimately alter their physiochemical properties.^27,28^ R. V. Gonçalves et al.^28^ systematically investigated the crystallization process of free-standing ATO NTs by annealing them at 750, 800, and 900 °C in air for 0.5 or 1 h. They observed the orthorhombic β-Ta_2_O_5_ phase for all of the annealed NT samples with minimal impact on the nanotubular morphology (Figure 16a,b). However, slight distortion in the nanotube structure was observed for samples treated at 800 °C for 1 h. It was further indicated that annealing above 800 °C may cause significant deformation in NT walls. XPS studies revealed the complete removal of S and F in the heat treated Ta_2_O_5_ NTs compared to the freshly prepared NT samples (6 and 5 at. % of S and F) (Figure 16c). An increase in the O/Ta ratio was also observed in all of the annealed NTs compared to the untreated ones. However, another study of Ta_2_O_5_ nanotubes prepared in NH_4_F containing electrolytes showed a significant amount of fluorine (4.15–6.36 at. %) in the nanostructures even after thermal treatment at 450 °C for 1 h in air.^44^ Through XPS studies, S. Singh et al.^97^ demonstrated that the fluoride content in free-standing anodic Ta_2_O_5_ layers can be reduced simply by washing them with water for a few seconds, as the tantalum oxyfluoride layer present in the outer layer of the pores/tubes is soluble in water.

Furthermore, various studies have highlighted that the crystallization temperature and thermal stability of anodic tantalum oxide strongly depend on the preparation conditions. It is anticipated that the introduction of dopants or impurities from the electrolyte can induce changes in the crystallization temperature and thermal stability of the anodic nanostructures.^50^ For example, anodic tantalum oxide nanostructures prepared in H_2_SO_4_ + HF electrolytes with additives, such as H_3_PO_4_, ethylene glycol, or dimethyl sulfoxide exhibited crystallization temperatures in the range of 300–550 °C.^57^ In contrast, free-standing NTs prepared in H_2_SO_4_ + HF electrolytes with additional additives showed a crystallization temperature above 750 °C.^27,42^ Similarly, several studies have investigated the impact of annealing temperature, and time on the crystallinity of anodic structures.^36,37,40,59^

M. M. Momeni et al.^36,37^ demonstrated that anodic tantalum oxide nanotubes obtained after 2 h of annealing in air at 400 °C are partially crystalline, displaying some XRD diffraction patterns of the Ta_2_O_5_ phase. M.A.A. Talip et al.^59^ observed XRD diffraction patterns of the orthorhombic Ta_2_O_5_ (JCPDS, No. 25–0922) for the nanotube samples annealed at 500 °C for 2 h in air. H. Yu et al.^26^ investigated the effect of annealing on the crystallinity of coral-like anodic tantalum oxide in air (550 °C, 3 h), observing diffraction peaks of β-Ta_2_O_5_ with an orthorhombic phase (JCPDS, No. 89–2843) in this case. Using Scherrer’s equation, they estimated the average crystallite size to be around 27.6 nm. It is noteworthy that high-temperature treatment above 1000 °C can induce a transition from β-Ta_2_O_5_ to α-Ta_2_O_5_ phase.^86,114^ In addition, post-treatment methods such as annealing ATOs in H_2_S and NH_3_ gas were performed to widen their applicability.^21,22,40^ P. Li et al. demonstrated the partial and complete conversion of ATO nanotube films to TaS_2_ nanotubes by annealing in H_2_S atmosphere at 625 °C.^22^ The XRD and electron diffraction studies revealed complete conversion to the TaS_2_ phase when treated under these conditions for 24 h. The TaS_2_ nanotubes produced in this study exhibited a higher superconductivity compared to bulk TaS_2_. S. Banerjee et al. synthesized TaON nanotubes by treating ATO nanotubes at 700 °C for 6 h in an NH_3_ atmosphere. The formation of the TaON phase was revealed through XRD and electron diffraction studies.^21^ In this case, the UV–vis absorption properties and band gap energy (2.07 eV) of TaON nanotubes were found to be more favorable for solar water splitting compared to untreated ATO nanotubes. Under AM 1.5 conditions in a 1 M KOH solution, it is demonstrated that TaON nanotubes generated a photocurrent density of 2.6 mA cm^–2^ at 0.5 V (vs Ag/AgCl), which was higher compared to ATOs (0.25 mA cm^–2^). Moreover, the photocatalytic activity of the TaON nanotubes was found to be stable for up to 2 h. Similarly, S. Xia et al. annealed nanoporous ATO films in an argon or air atmosphere for 2 h at 350, 450, and 550 °C.^40^ This study demonstrated that thermal annealing of anodic Ta_2_O_5_ in an inert atmosphere can lead to an oxygen deficiency in the metal oxide layer, which may be beneficial for Li-ion storage. The Ta_2_O_5_ films produced under post-treatment conditions in an argon atmosphere at 450 °C demonstrated a high lithium capacity of ∼480 mAh g^–1^ with excellent cycling stability (up to 8000 cycles) compared to Ta_2_O_5_ films treated under similar conditions in an air atmosphere. They hypothesized that a high level of oxygen defects with an amorphous nature is beneficial in this case. These results underscore the significant role of thermal treatment conditions in shaping the crystalline phases of anodic Ta_2_O_5_, with additional effects on the morphology and elemental composition. Details of the annealing conditions and their effects on the crystalline phases of anodic tantalum oxide can be found in Table 5.

**Table 5:** Morphology, Annealing Conditions, and Crystal Structures of the Resulting ATO Layers

Oxide morphology	Oxide layer thickness	Annealing temperature (°C)	Annealing atmosphere	Annealing time (min)	Phases after annealing	Ref
Compact	200–5000 Å	500–800	Vacuum	60–240	NA	(70)
Compact	NA	450	High vacuum (10^–5^ Torr)	10	NA	(95)
Compact	NA	500–700	Air	0.16	NA	(81)
Compact	NA	300–400	Air	NA	NA	(94)
Compact	Up to 250 nm	300–800	Air	30–180	Orthorhombic β-Ta_2_O_5_	(41)
Nanopores	150 nm	550	Air	180	Orthorhombic Ta_2_O_5_	(26)
Nanopores	NA	350–550	Ar	120	Oxygen deficient Ta_2_O_5_ Orthorhombic Ta_2_O_5_	(40)
Nanopores	NA	290	Air	10	Hexagonal Ta_2_O_5_	(29)
Nanopores	NA	400	O_2_	120	Ta_2_O_5_	(36)
Nanopores and Nanotubes	NA	450	Ar	60	Ta_2_O_5_	(84)
Nanorods	NA	1000	NH_3_	120	Ta_3_N_5_	(23, 86)
Nanorods	NA	1000	NH_3_	120	Ta_3_N_5_	(23)
Nanotubes	NA	300 and 400	Air	180	Ta_2_O_5_	(57)
Nanotubes	NA	700	NH_3_	360	TaON	(21)
Nanotubes	NA	750–800	Ar	720	Orthorhombic Ta_2_O_5_	(42)
Nanotubes	NA	625	H_2_S	1440	2H-TaS_2_	(22)
Nanotubes	NA	550–800	Air	10–60	Orthorhombic Ta_2_O_5_	(27)
Nanotubes	NA	800–1000	Air	60	Orthorhombic Ta_2_O_5_	(28)
Nanotubes	NA	1000	NH_3_	120	Ta_3_N_5_	(24)
Nanotubes	NA	1000	NH_3_	120	Ta_3_N_5_	(25)
Nanotubes	NA	800	Air	60	Ta_2_O_5_	(34)
Nanotubes	NA	450	Air	60	Orthorhombic Ta_2_O_5_	(31)
Nanotubes	2 μm	400	Air	120	Ta_2_O_5_	(37)
Nanotubes	NA	440 and 550	Air	60–180	Orthorhombic Ta_2_O_5_	(58)
Nanotubes	NA	500	Air	120	Orthorhombic Ta_2_O_5_	(59)
Nanotubes	1.68–2.19 μm	450–900	Air, NH_3_, N_2_, or H_2_	60	Ta_2_O_5_, Ta_4_O	(51)
Nanotubes	NA	500	Air	120	Ta_2_O_5_	(60)
Ta_2_O_6.6_S_0.7_ Nanotubes	NA	785 and 855	Air	60	Orthorhombic β-Ta_2_O_5_	(50)

**Scheme 1:**
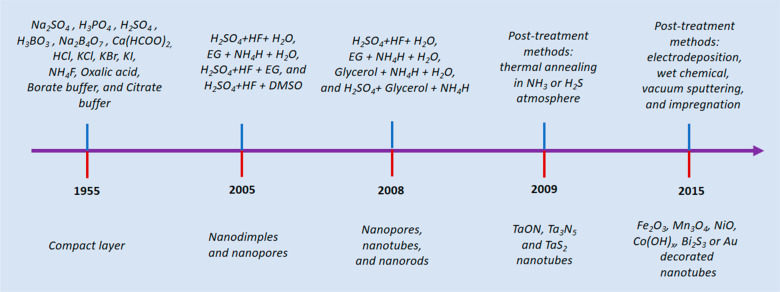
Chronology of the Fabrication of ATO Structures and Their Post-treatment Methods

Apart from this, post-treatment also includes decoration of secondary nanoparticles of metals, metal oxides, metal hydroxides, and metal sulfides (e.g., Fe_2_O_3_, Mn_3_O_4_, NiO, Co(OH)_x_, Bi_2_S_3_, and Au) on ATO or its derived materials by various multistep methods.^24,34,35,44,51,114^ The chronology of the fabrication of ATO structures and their post-treatment conditions are presented in Scheme 1 for a better understanding of these methods. Furthermore, some notable examples of these strategies to enhance their performance are discussed in the next section.

## 7 Applications of Anodic Tantalum Oxides

The size, morphology, crystallinity, and impurity content of ATO or ATO-derived materials can significantly influence their physicochemical properties.^18,30−34,40,46,55,56,60,64,68,77,78,90,82,84,112−115^ Although ATO-based capacitors have been investigated since the 1980s/1990s,^87,93,94^ in the last two decades, efforts have also been made to explore the potential of ATO-based materials in the fields of memristive devices, photocatalysis, electrocatalysis, corrosion resistant coatings, and biomedical applications.^46,55,56,60,64,68,77,78,90,82,84,112−115^ In this section, we summarize some notable physiochemical properties and applications of ATOs or their derived materials.

### 7.1 Capacitors and Memristive Devices

**Figure 17:**
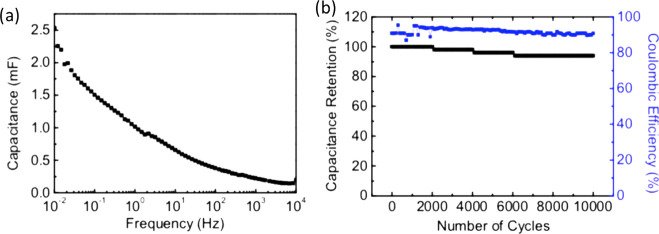
(a) Electrochemical capacitance vs frequency for Ta_2_O_5_/Fe_2_O_3_ NTs layers. (b) Capacitance retention and Coulombic efficiency during cycling tests for the material measured at 1 mA cm^–2^ for 10000 cycles. Reproduced with permission from ref (115). Copyright 2014 American Chemical Society.

Capacitors based on the Ta or Ta_2_O_5_ have been extensively investigated and find applications in many electronic devices including mobile phones, computers, and space equipment, etc.^87,93,94,115^ M. A. Mohammed et al.^94^ studied the capacitance–voltage characteristics of Ta_2_O_5_ films synthesized by multiple methods, such as anodization, thermal annealing, and ion-implantation-based enhanced thermal oxidation. It was demonstrated that the room temperature capacitance of anodic Ta_2_O_5_ films increased with applied bias after annealing the sample at 500 °C. The enhanced capacitance behavior was attributed to a structural transformation of Ta_2_O_5_ from an amorphous to a partially polycrystalline state. More recently, a Ta_2_O_5_/Fe_2_O_3_ composite, based on 3D nanotube arrays, has been explored as an electrode material for supercapacitor application.^115^ In this case, Fe_2_O_3_ nanoparticles were anchored on the walls of Ta_2_O_5_ nanotubes using a wet chemical deposition method, and the structural arrangement was believed to efficiently relieve strain during the cycles. The Ta_2_O_5_/Fe_2_O_3_ nanocomposite arrays exhibited excellent capacitive characteristics with remarkable stability over 10000 cycles (Figure 17).

In addition to this, efforts have been dedicated to explore the memristive behavior of anodic Ta_2_O_5_ films.^11,67,80^ M. Diamanti et al.^67^ conducted a systematic investigation, comparing the memristive properties of anodic Ti, Ta, and Nb oxide films formed in phosphoric acid at different potentials (10, 20, 25, and 30 V). The oxide film thickness was controlled in the range 30–200 nm. It was observed that the thickness of the oxide films plays a major role in the memristive switching properties. The synthesized Nb_2_O_5_, TiO_2_, and Ta_2_O_5_ oxide films with thicknesses in the range of a few tens of nanometers, demonstrated superior memristive performance compared to thicker films. Similarly, I. Zrinski et al.^80^ studied memristive properties of the Hf–Ta system, wherein a 15 nm thick anodic oxide layer was obtained by anodization of the sputter deposited Ta–Hf layer in a citrate buffer solution.

### 7.2 Catalytic Activity and Degradation of Chemical Substances

**Figure 18:**
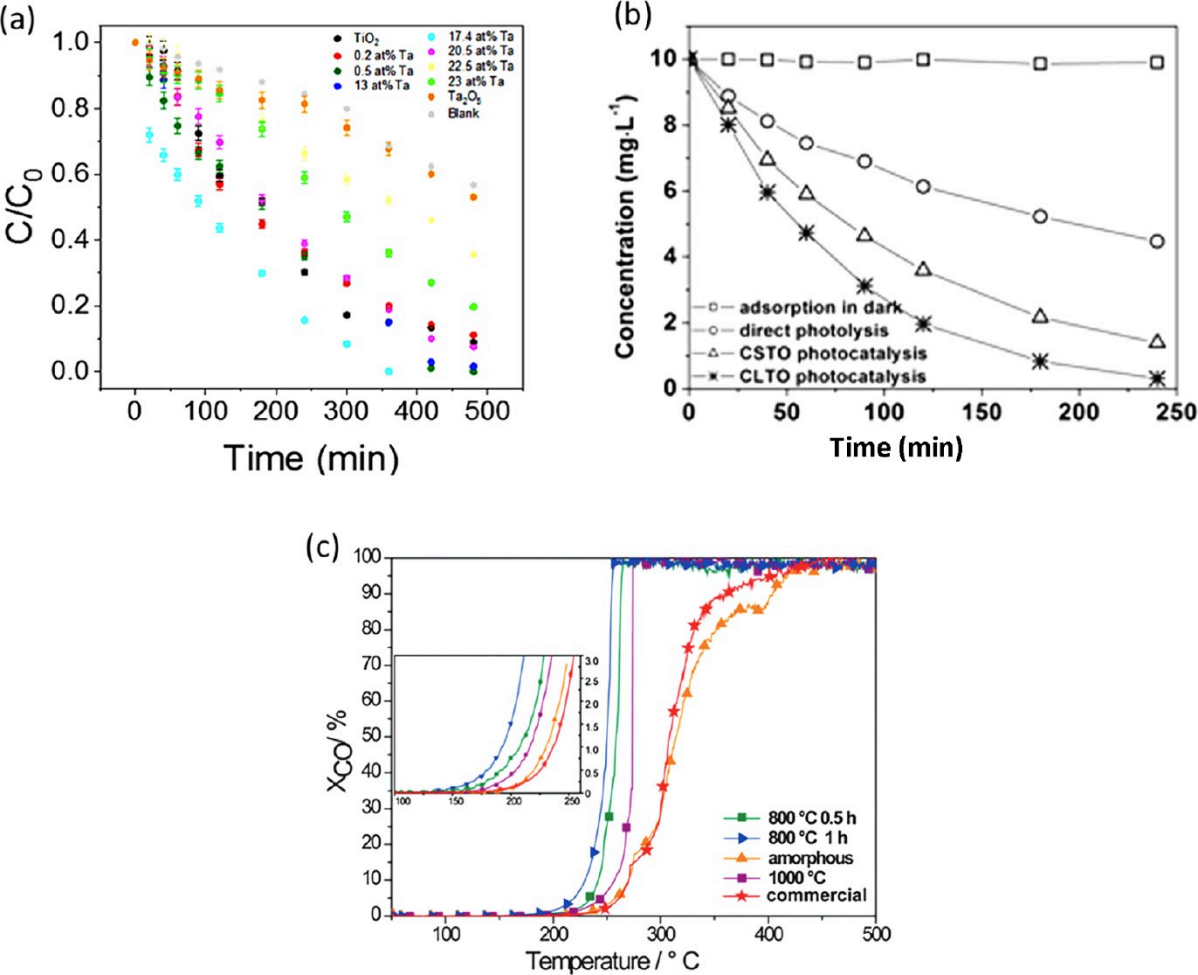
(a) Degradation of MB for different anodized Ti–Ta substrates with various Ta at % loadings. A 320–480 nm filter was applied to the light source. Reproduced with permission from ref (84). Copyright 2019 American Chemical Society. (b) Variation of phenol concentration with time during degradation tests at compact surface tantalum oxide (CSTO) and coral like tantalum oxide (CLTO) photocatalysts. A 450 W high-pressure mercury lamp served as the UV light source. Reproduced with permission from ref (26). Copyright 2013 PLOS. (c) CO conversion as a function of temperature over commercial Ta_2_O_5_, freestanding amorphous Ta_2_O_5_ nanotubes, and Ta_2_O_5_ nanotubes heat-treated at various temperatures. Reproduced with permission from ref (28). Copyright 2014 Royal Society of Chemistry.

The *n*-type semiconductor properties of nanostructured ATOs, with a band gap in the range of 3.8–4.5 eV, allow it to generate electron–hole pairs upon exposure to photons with energies equals to or higher than its band gap.^21,26,27,36^ Consequently, this material can serve as a catalyst for various photochemical and photoelectrochemical reactions. M. M. Momeni et al.^36^ demonstrated a photocatalytic degradation of methylene blue (MB) on anodic tantalum oxide nanotube films formed under varying anodizing conditions. The high photocatalytic activity observed for the nanotube films (2 μm thick) is attributed to the large surface area and semiconducting properties of the material, believed to enhance the adsorption efficiency of light and facilitate high diffusion rates of reactants and products. In another study, A. Merenda et al.^84^ designed sub-10 nm surface pore size titanium–tantalum oxide substrates by electrochemical oxidation of a Ta–Ti alloy matrix. They further adjusted the band gap of the material by altering the Ta/Ti surface ratio, demonstrating that a composite with a minimum band gap exhibits higher activity in the degradation of MB under visible light compared to pure TiO_2_ (Figure 18a).

H. Yu et al.^26^ developed nanoporous tantalum oxide films with a coral-like morphology for the photocatalytic decomposition of phenol. In this study, the material with a band gap of 3.8 eV and pore diameter of 30–50 nm showed high efficiency in the photocatalytic decomposition of phenol compared to a compact oxide layer (Figure 18b). In another work, ATO nanotubes decorated with Bi_2_S_3_ quantum dots (QDs), with external and internal nanotube diameters of 41 and 19 nm, o and an oxide thickness of 1.27 μm, demonstrated a high photocatalytic activity toward toluene degradation. About 99% of toluene (200 ppm) was degraded after 5 min of UV–Vis irradiation.^51^

Moreover, ATOs have also exhibited catalytic activity for gaseous phase reactions. R.V. Gonçalves et al.^28^ compared the catalytic CO oxidation activity of commercial Ta_2_O_5_ with freestanding ATO nanotubes treated under various annealing conditions (Figure 18c). In this study, thermally treated ATO nanotubes showed higher activity compared to amorphous and commercial Ta_2_O_5_.

### 7.3 Energy Related Applications (Photoelectrochemical Catalysis and H_2_ Generation)

**Figure 19:**
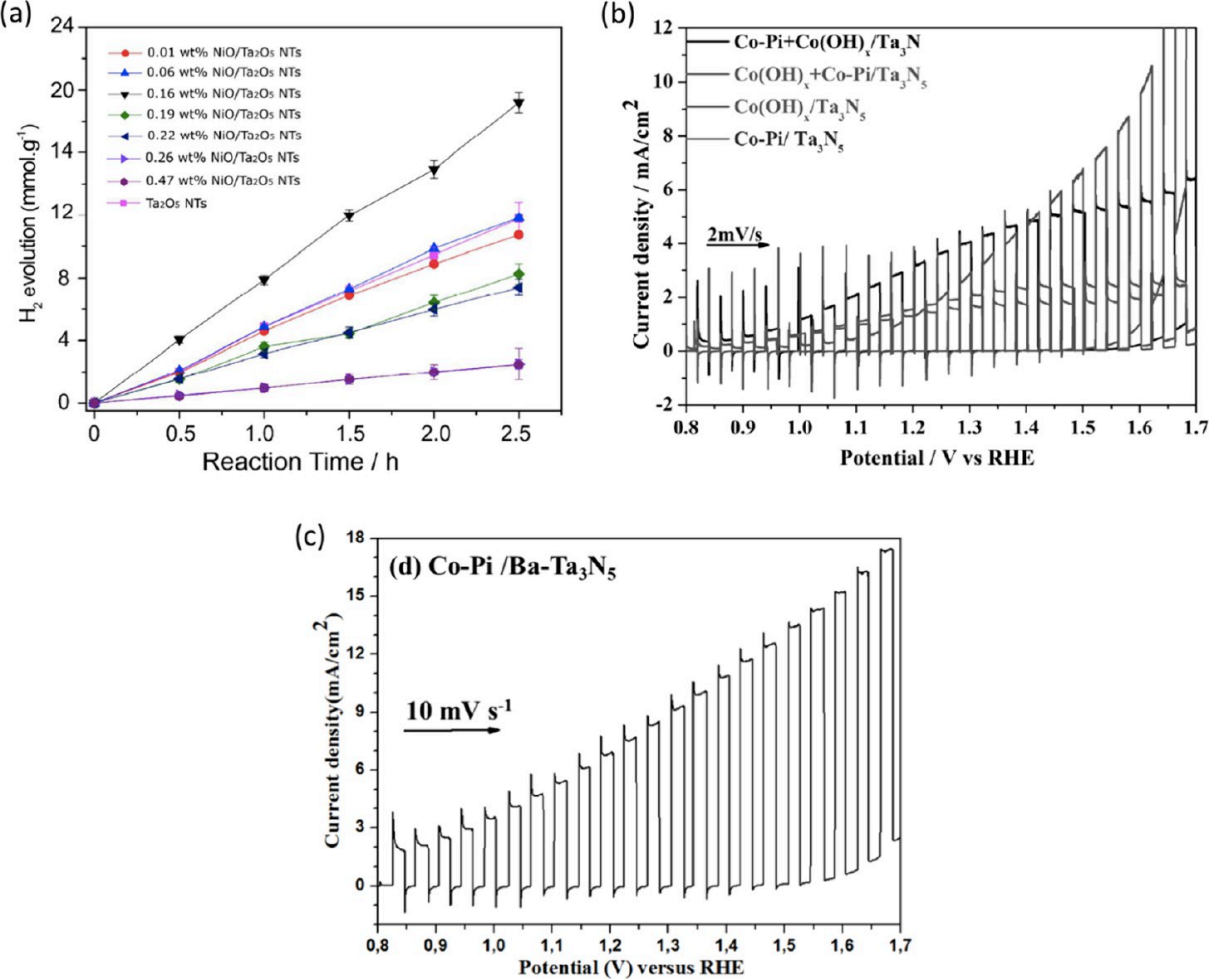
(a) Hydrogen evolution for pure Ta_2_O_5_ NTs and NTs with different concentrations of NiO NPs on their surface in an H_2_O/ethanol (4:1 vol.) solution. A xenon lamp of 300 W and a light intensity of 400 mW cm^–2^ was utilized for this study. Reproduced with permission from ref (34). Copyright 2017 American Chemical Society. (b) Photocurrent–potential curves of open Ta_3_N_5_ nanotubes loaded with different cocatalysts (Co(OH)_x_, Co-Pi, Co(OH)_x_ + Co-Pi, and Co-Pi + Co(OH)_x_) under chopped AM 1.5 G simulated sunlight in a 1 M KOH solution (pH = 13.7) at a scan rate of 2 mV s^–1^. Reproduced with permission from ref (24). Copyright 2015 Elsevier. (c) Current–potential curves for Co-Pi/Ba–Ta_3_N_5_ photoelectrode under chopped AM 1.5 G simulated sunlight in a 1 M KOH solution (pH = 13.7) at a scan rate of 10 mV s^–1^. Reproduced with permission from ref (25). Copyright 2015 Elsevier.

In the previous decade, Ta_2_O_5_ or modified Ta_2_O_5_, has been extensively investigated as a photocatalyst or photoelectrochemical catalyst for water splitting under UV irradiation/solar light conditions.^21,23−25,27,34^ R.V. Gonçalves et al.^27^ elucidate the crucial role of annealing conditions on the photocatalytic hydrogen generation activity of freestanding anodic Ta_2_O_5_ NTs. The study demonstrated that high crystallinity, high surface area, and low surface contamination in the ATO NTs can enhance their photocatalytic activity for hydrogen production. Specifically, ATO NTs treated at 800 °C for 1 h in air exhibited 1.6 times higher activity compared to ATO NTs treated at 800 °C for 0.5 h. The same research group further developed a heterogeneous structure of anodic ATO NTs decorated with *p*-type NiO nanoparticles as a photocatalyst for hydrogen evolution from a water–ethanol solution.^34^ Ni nanoparticles were deposited onto the ATO NTs by direct-current (dc) magnetron sputtering in a vacuum. The duration of sputtering was varied from 5 to 180 s to increase the Ni loading from 0.01 to 0.47 wt %. After deposition, the heterostructures were calcined in air for 2 h at 500 °C to convert the deposited Ni to its oxide form (NiO). In this case, the ATO NTs loaded with 0.16 wt % of NiO showed higher hydrogen evolution activity (up to 7.7 ± 0.3 mmol h^–1^ g^–1^) compared to pure ATO NTs (4.9 ± 0.3 mmol h^–1^ g^–1^) (Figure 19a). It is suggested that the electrons from the conduction band of NiO can migrate to Ta_2_O_5_ during water reduction, and photogenerated holes from Ta_2_O_5_ can move to the valence band of NiO to assist the ethanol oxidation reaction. Additionally, several studies have demonstrated that the transformation of anodic Ta_2_O_5_ to Ta_3_N_5_ can reduce and shift the band gap energy to 2.1 eV, providing to be beneficial for solar spectrum (<600 nm) driven PEC water splitting.^21,23−25^

For example, S. Grigorescu et al.^24^ converted ATO NTs into Ta_3_N_5_ NTs by nitridation in an ammonia atmosphere and modified the surface of Ta_3_N_5_ NTs with cobalt phosphate (Co–Pi) or Co(OH)_x_ or combination of Co–Pi + Co(OH)_x_. In this case, Ta_3_N_5_–Co(OH)_x_ was prepared by depositing Co(OH)_x_ on Ta_3_N_5_ NTs by a simple wet chemical method, while Ta_3_N_5_–Co–Pi was prepared by the electrodeposition of Co–Pi from a Co(NO)_3_ + potassium phosphate electrolyte bath. The Ta_3_N_5_ photoanode modified with both Co–Pi and Co(OH)_x_ (i.e., Co–Pi + Co(OH)_x_/Ta_3_N_5_) exhibited an enhanced photocurrent density up to 3.1 mA cm^–2^ at 1.23 V vs RHE compared to Ta_3_N_5_ /Co–Pi and Ta_3_N_5_ /Co(OH)_x_ photoelectrodes (Figure 19b). Z. Su at al.^25^ also investigated PEC water splitting activity of Ta_3_N_5_/Co–Pi and Ta_3_N_5_/Co(OH)_x_ photoelectrodes. They observed remarkable photocurrents of 5.9 mA cm^–2^ at 1.23 V_RHE_ and 12.9 mA cm^–2^ at 1.59 V_RHE_ for Co–Pi/Ta_3_N_5_. Additionally, they synthesized Ba-doped Ta_3_N_5_ and studied the PEC water splitting activity of Co–Pi/Ba–Ta_3_N_5_, which showed an enhanced photocurrent density of 7.5 mA cm^–2^ at 1.23 V_RHE_ (Figure 19c). Beyond nanotube structures, Y. Li et al.^86^ successfully synthesized Co–Pi modified nanorod structures of Ta_3_N_5_ and investigated their PEC water splitting activity in a 0.5 M K_2_HPO_4_ electrolyte. This material achieved a maximum solar conversion efficiency of 0.36% and a photocurrent density of 1.5 mA cm^–2^ at 1 V versus RHE under an AM of 1.5 G.

### 7.4 Anticorrosion Coatings

**Figure 20:**
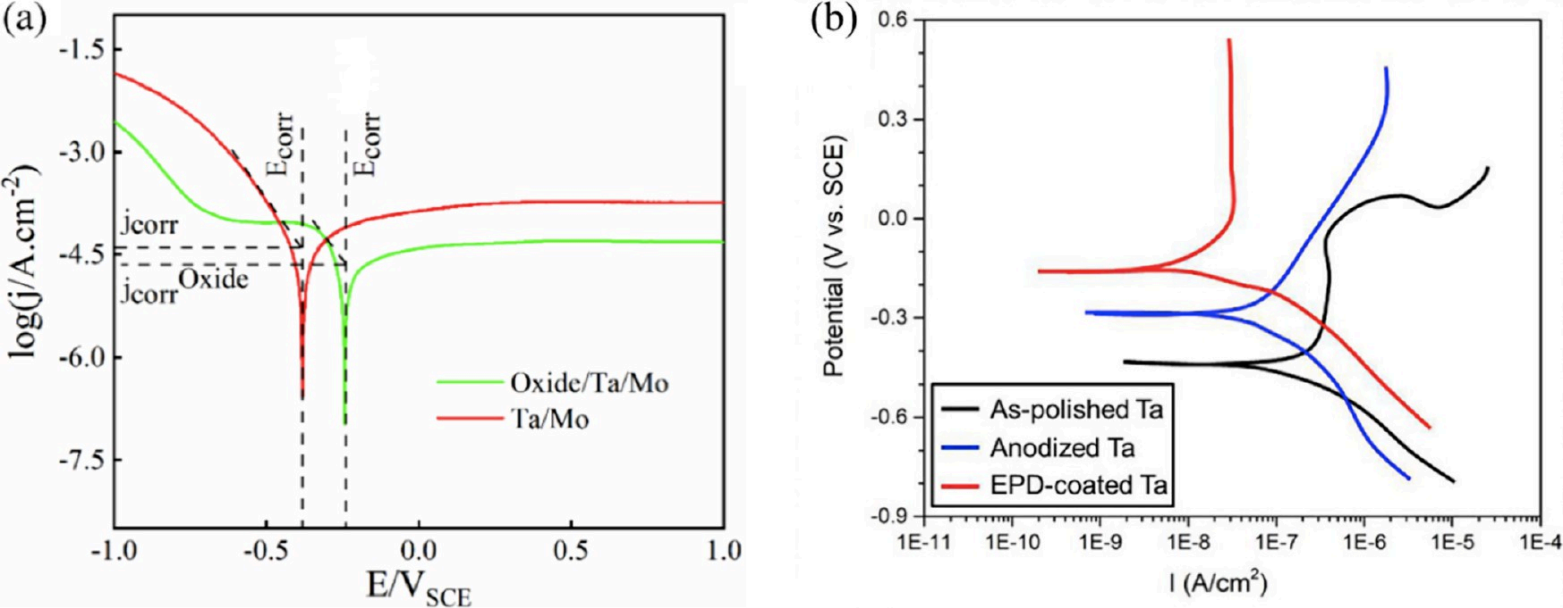
Potentiodynamic polarization curves of (a) Ta/Mo and oxide/Ta/Mo samples in a 3.5 wt % NaCl solution at 25 °C. Reproduced with permission from ref (83). Copyright 2021 Elsevier. (b) As-polished tantalum, anodic, and EPD coated anodic tantalum in phosphate-buffered saline (PBS) containing 150 mM H_2_O_2_ with pH 5.2 adjusted with HCl (37%). Reproduced with permission from ref (44). Copyright 2022 Elsevier.

Tantalum or tantalum-based materials are recognized for their stability under acidic conditions, making them excellent candidates for anticorrosion coatings.^31,44,83^ J. Hou et al.^83^ investigated corrosion-resistance behavior of a 150 nm thick Ta_2_O_5_ coating produced by anodic oxidation of a Ta/Mo matrix. In a 3.5% NaCl solution, the anodic Ta_2_O_5_/Ta/Mo matrix displayed a higher corrosion potential (*E*_corr_ = 0.140 V) and a lower corrosion current density (*j*_corr_ = 0.009 A cm^–2^) compared to that of the pure Ta/Mo sample (Figure 20a). SEM studies indicated an improvement in the compactness of the Ta_2_O_5_ coating on the Ta/Mo matrix, which is believed to be responsible for enhanced resistance against Cl^–^ ions. More recently, A. Bordbar-Khiabani et al.^44^ designed Mn_3_O_4_ NPs coated ATO films for the corrosion resistance in a biochemical environment (i.e., simulated inflammatory conditions around metallic implants in the human body).^44^ In this case, the Mn_3_O_4_ NPs were coated through the electrophoretic deposition (PED) method, utilizing anodized-tantalum as the cathode. The TEM studies confirmed the deposition of spherically shaped Mn_3_O_4_ NPs on the ATO substrate. They observed improved corrosion resistance activity of Mn_3_O_4_ NPs coated films with a positive shift in corrosion potential (*E*_corr_) compared to that of anodized and polished Ta (Figure 20b).

### 7.5 Medical Applications

**Figure 21:**
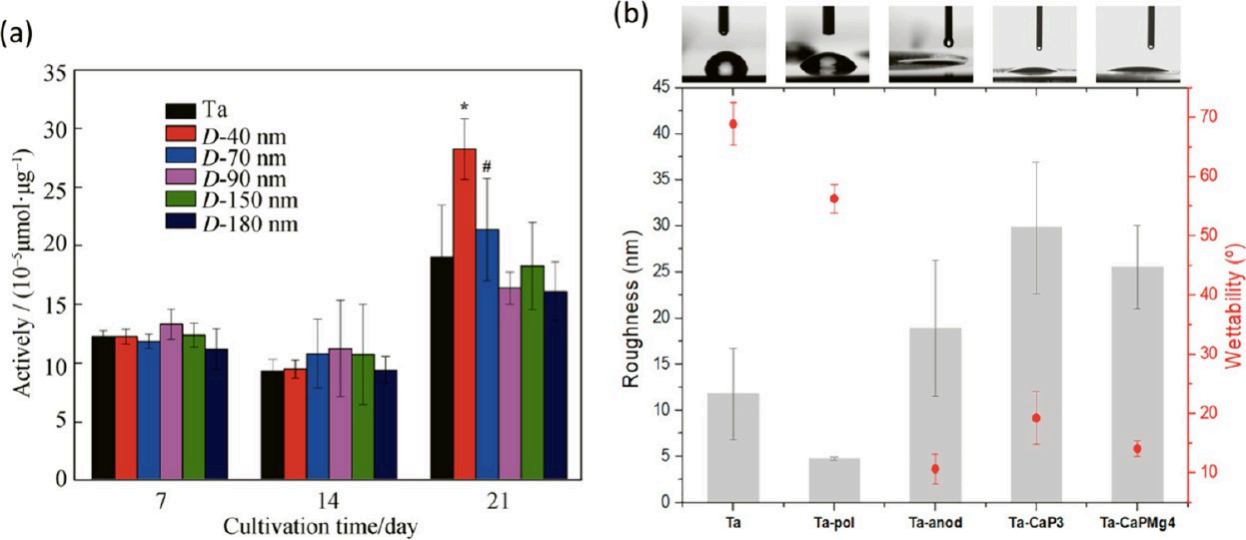
(a) Alkaline phosphatase (ALP) activity tests for culturing MC-3T3-E1 cells for 7, 14, and 21 days on nanodimpled surfaces with different diameters formed on tantalum metal (activity values of all groups in 21 days are significantly different from those of the corresponding groups in 7 and 14 days); ∗ indicates a significant difference between the 40 nm group and all other groups with the same cell culture time; # indicates a significant difference between the 70 nm group and all other groups with the same cell culture time. Reproduced with permission from ref (46). Copyright 2019 Springer. (b) Relationship between roughness (column plot) and wettability (scatter plot) for each surface modification along with photographs of wetting behavior of surface with a Milli-Q ultrapure water drop where Ta-CaP3 is synthesized by 2 step anodization in 0.18 M calcium acetate (CaA) + 0.12 M β-glycerolphosphate (β-GP) at 100 V for 30 min and Ta-CaPMg4 is synthesized by 2 step anodization in 0.18 M CaA + 0.12 M β-GP + 0.08 M magnesium acetate (MgA) at 80 V for 30 min. Reproduced with permission from ref (33). Copyright 2019 Elsevier.

As mentioned earlier, tantalum has the ability to form a spontaneous passive oxide film on the surface, providing protection against harsh acidic and biological environments.^31,46,83^ Therefore, tantalum, tantalum oxide, or its alloys with hierarchical micro/nanostructured surfaces hold great potential for orthopedic and dental implant applications. For example, J. W. Ma et al.^46^ designed an anodic tantalum surface with a nanodimpled morphology. In this study, the dimple diameter ranged from 40 to 180 nm, and the dimples exhibited a hydrophilic nature. The researchers observed an increase in the dimple diameter with an increase in the anodization potential in H_2_SO_4_ + HF electrolytes. In the in vitro studies, they demonstrated a significant promotion in the adhesion, proliferation, and differentiation of MC-3T3-E1 cells on nanodimpled structures compared to a pure Ta surface. It was further shown that the Ta surface with a small dimple size (40 nm) is more beneficial for cell proliferation and osteogenic differentiation compared to the surface with a larger dimple size (Figure 21a). Recently, L. Fialho et al.^33^ implemented strategies for incorporating osteoconductive elements, such as Ca, P, and Mg into the surface of anodic tantalum oxide layers, aiming to enhance the bioactivity and bone implant applications. They designed a bone-like structure of porous tantalum oxide using an anodic oxidation process. The surface oxide layer was doped with Ca, P, and Mg through a cathodic polarization or anodization method in an electrolyte containing Ca, P, and Mg precursors. Contact angle studies revealed improved hydrophilicity for both doped and undoped anodic tantalum oxide structures compared to the pristine or polished Ta substrate (Figure 21b). Additionally, the doped anodic tantalum oxide structures showed higher surface roughness compared to that of undoped anodic tantalum oxide. The researchers speculated that the modified surface properties of these materials could promote osseointegration processes and remineralization of dental implants.

## 8 Conclusions and Outlooks

In this comprehensive review, we delve into the recent advancements in the synthesis and applications of anodic tantalum oxide-based materials. The anodization process has emerged as a versatile method for fabricating tantalum oxides with diverse morphologies, including a compact oxide layer and nanoporous, nanotubular, nanocoral, and nanodimple structures. We meticulously present and discuss the anodization conditions governing the fabrication of these oxide forms, shedding light on the intricacies of the process. Notably, we emphasize various anodization parameters crucial for controlling the morphology of nanoporous/nanotubular structures, such as pore size and tube length, and we explore the physical characterization methods used for detailed analyses.

Our review extends to the elucidation of the growth mechanism underlying anodic nanostructures, providing insights into the chemical processes that dictate their formation. By summarizing the latest research findings, we contribute to advancing the understanding of the chemistry governing the synthesis of anodic tantalum oxide nanostructures. Additionally, we delve into the significance of the annealing method as a means to enhance the physiochemical properties of anodic tantalum oxides. This section provides a comprehensive overview of the strategies employed for annealing, offering valuable insights into optimizing the properties of these materials.

Furthermore, our review encompasses the modifications applied to anodic tantalum oxides, including surface modifications and/or doping with various metals, nonmetals, and metal oxides. Notably, surface modifications have emerged as a promising strategy to enhance photochemical and photoelectrochemical activities of anodic tantalum oxides. We thoroughly discuss the methods and outcomes of such modifications, highlighting their impact on the performance of tantalum oxide-based materials. As we navigate through these discussions, it becomes evident that surface modification holds tremendous potential for elevating the functionalities of anodic tantalum oxides. The nuanced exploration of the interplay between surface modifications and the resulting improvements in photochemical and photoelectrochemical activities underscores the significance of this approach.

In our closing remarks, we posit that future investigations in two key directions will be pivotal. First, we advocate for a concerted focus on further exploration and refinement of surface modifications for anodic tantalum oxides. This avenue presents a promising trajectory for enhancing the overall performance and applicability of these materials in various technological domains. Second, we underscore the importance of manipulating anodizing parameters to engineer novel nanostructures, such as nanowires, nanorods, nanospheres, and more. This strategic manipulation of anodization conditions offers an exciting platform for designing tantalum oxide-based materials with vastly improved properties. The exploration of novel nanostructures not only expands the material landscape but also opens new avenues for tailoring tantalum oxide materials to meet specific application requirements.

In conclusion, this review encapsulates a holistic overview of recent developments in the synthesis and applications of anodic tantalum oxide-based materials. From elucidating anodization conditions and growth mechanisms to exploring surface modifications and doping strategies, we provide a comprehensive resource for researchers and practitioners in the field. We anticipate that this review will inspire further innovations and discoveries in the pursuit of advanced materials with enhanced properties and diverse applications.

## Abbreviations

ATO: anodic tantalum oxides
TMO: transition metal oxides
CVD: chemical vapor deposition
PEC: photoelectrochemical
D: diameter
L: length
NT: nanotubes
AT: anodizing temperature
RT: room temperature
EG: ethylene glycol
DMSO: dimethyl sulfoxide
MB: methylene blue
SIMS: secondary ion mass spectroscopy
QDs: quantum dots
CLTO: coral like tantalum oxide
CSTO: compact surface tantalum oxide
RHE: reversible hydrogen electrode
XPS: X-ray photo electron spectroscopy
TEM: transmission electron microscopy
SEM: scanning electron microscopy
XRD: X-ray diffraction
PED: electrophoretic deposition
PBS: phosphate-buffered saline
ALP: Alkaline phosphatase
β-GP: β-glycerolphosphate
CaA: calcium acetate
MgA: magnesium acetate
